# Diagnostic Performance of Cortical Lesions and the Central Vein Sign in Multiple Sclerosis

**DOI:** 10.1001/jamaneurol.2023.4737

**Published:** 2023-12-11

**Authors:** Alessandro Cagol, Rosa Cortese, Muhamed Barakovic, Sabine Schaedelin, Esther Ruberte, Martina Absinta, Frederik Barkhof, Massimiliano Calabrese, Marco Castellaro, Olga Ciccarelli, Sirio Cocozza, Nicola De Stefano, Christian Enzinger, Massimo Filippi, Maciej Jurynczyk, Pietro Maggi, Nima Mahmoudi, Silvia Messina, Xavier Montalban, Jacqueline Palace, Giuseppe Pontillo, Anne-Katrin Pröbstel, Maria A. Rocca, Stefan Ropele, Àlex Rovira, Menno M. Schoonheim, Piotr Sowa, Eva Strijbis, Mike P. Wattjes, Maria Pia Sormani, Ludwig Kappos, Cristina Granziera

**Affiliations:** 1 Translational Imaging in Neurology Basel, Department of Biomedical Engineering, University Hospital Basel and University of Basel, Basel, Switzerland; 2 Department of Neurology, University Hospital Basel, Switzerland; 3 Research Center for Clinical Neuroimmunology and Neuroscience Basel, University Hospital Basel and University of Basel, Basel, Switzerland; 4 Department of Health Sciences, University of Genova, Genova, Italy; 5 Department of Medicine, Surgery and Neuroscience, University of Siena, Siena, Italy; 6 Queen Square Multiple Sclerosis Centre, Department of Neuroinflammation, University College London Queen Square Institute of Neurology, Faculty of Brain Sciences, University College London, London, United Kingdom; 7 Department of Clinical Research, University Hospital Basel, University of Basel, Basel, Switzerland; 8 Medical Image Analysis Center, Basel, Switzerland; 9 Institute of Experimental Neurology, Division of Neuroscience, Vita-Salute San Raffaele University and Istituto di Ricovero e Cura a Carattere Scientifico, San Raffaele Scientific Institute, Milan, Italy; 10 Queen Square Institute of Neurology and Centre for Medical Image Computing, University College London, United Kingdom; 11 Multiple Sclerosis Center Amsterdam, Radiology and Nuclear Medicine, Vrije Universiteit Amsterdam, Amsterdam Neuroscience, Amsterdam University Medical College VUMC, Amsterdam, the Netherlands; 12 Department of Neurosciences, Biomedicine and Movement Sciences, University of Verona, Verona, Italy; 13 Department of Information Engineering, University of Padova, Padova, Italy; 14 National Institute for Health and Care Research (NIHR) University College London Hospitals Biomedical Research Centre, London, United Kingdom; 15 Departments of Advanced Biomedical Sciences and Electrical Engineering and Information Technology, University of Naples Federico II, Naples, Italy; 16 Department of Neurology, Medical University of Graz, Graz, Austria; 17 Division of Neuroradiology, Vascular and Interventional Radiology, Department of Radiology, Medical University of Graz, Graz, Austria; 18 Neuroimaging Research Unit, Division of Neuroscience, Istituto di Ricovero e Cura a Carattere Scientifico, San Raffaele Scientific Institute, Milan, Italy; 19 Neurology Unit, Istituto di Ricovero e Cura a Carattere Scientifico, San Raffaele Scientific Institute, Milan, Italy; 20 Neurorehabilitation Unit, Istituto di Ricovero e Cura a Carattere Scientifico, San Raffaele Scientific Institute, Milan, Italy; 21 Neurophysiology Service, Istituto di Ricovero e Cura a Carattere Scientifico, San Raffaele Scientific Institute, Milan, Italy; 22 Vita-Salute San Raffaele University, Milan, Italy; 23 Department of Clinical Neurology, Nuffield Department of Clinical Neurosciences, University of Oxford, Oxford, United Kingdom; 24 Laboratory of Brain Imaging, Neurobiology Center, Nencki Institute of Experimental Biology, Polish Academy of Sciences, Warsaw, Poland; 25 Department of Neurology, Cliniques Universitaires Saint-Luc, Université Catholique de Louvain, Brussels, Belgium; 26 Neuroinflammation Imaging Lab, Institute of Neuroscience, Université catholique de Louvain, Brussels, Belgium; 27 Department of Diagnostic and Interventional Neuroradiology, Hannover Medical School, Hannover, Germany; 28 Multiple Sclerosis Centre of Catalonia, Department of Neurology-Neuroimmunology, Hospital Universitari Vall d’Hebron, Universitat Autònoma de Barcelona, Barcelona, Spain; 29 Division of Neurology, St Michael’s Hospital, University of Toronto, Toronto, Ontario, Canada; 30 Departments of Biomedicine and Clinical Research, University Hospital of Basel and University of Basel, Basel, Switzerland; 31 Section of Neuroradiology, Department of Radiology, Hospital Universitari Vall d’Hebron, Universitat Autònoma de Barcelona, Barcelona, Spain; 32 Multiple Sclerosis Center Amsterdam, Anatomy and Neurosciences, Vrije Universiteit Amsterdam, Amsterdam Neuroscience, Amsterdam University Medical College VUMC, Amsterdam, the Netherlands; 33 Division of Radiology and Nuclear Medicine, Oslo University Hospital, Oslo, Norway; 34 Multiple Sclerosis Center Amsterdam, Neurology, Vrije Universiteit Amsterdam, Amsterdam Neuroscience, Amsterdam University Medical College VUMC, Amsterdam, the Netherlands; 35 Istituto di Ricovero e Cura a Carattere Scientifico, Ospedale Policlinico San Martino, Genova, Italy

## Abstract

**Importance:**

Multiple sclerosis (MS) misdiagnosis remains an important issue in clinical practice.

**Objective:**

To quantify the performance of cortical lesions (CLs) and central vein sign (CVS) in distinguishing MS from other conditions showing brain lesions on magnetic resonance imaging (MRI).

**Design, Setting, and Participants:**

This was a retrospective, cross-sectional multicenter study, with clinical and MRI data acquired between January 2010 and May 2020. Centralized MRI analysis was conducted between July 2020 and December 2022 by 2 raters blinded to participants’ diagnosis. Participants were recruited from 14 European centers and from a multicenter pan-European cohort. Eligible participants had a diagnosis of MS, clinically isolated syndrome (CIS), or non-MS conditions; availability of a brain 3-T MRI scan with at least 1 sequence suitable for CL and CVS assessment; presence of T2-hyperintense white matter lesions (WMLs). A total of 1051 individuals were included with either MS/CIS (n = 599; 386 [64.4%] female; mean [SD] age, 41.5 [12.3] years) or non-MS conditions (including other neuroinflammatory disorders, cerebrovascular disease, migraine, and incidental WMLs in healthy control individuals; n = 452; 302 [66.8%] female; mean [SD] age, 49.2 [14.5] years). Five individuals were excluded due to missing clinical or demographic information (n = 3) or unclear diagnosis (n = 2).

**Exposures:**

MS/CIS vs non-MS conditions.

**Main Outcomes and Measures:**

Area under the receiver operating characteristic curves (AUCs) were used to explore the diagnostic performance of CLs and the CVS in isolation and in combination; sensitivity, specificity, and accuracy were calculated for various cutoffs. The diagnostic importance of CLs and CVS compared to conventional MRI features (ie, presence of infratentorial, periventricular, and juxtacortical WMLs) was ranked with a random forest model.

**Results:**

The presence of CLs and the previously proposed 40% CVS rule had a sensitivity, specificity, and accuracy for MS of 59.0% (95% CI, 55.1-62.8), 93.6% (95% CI, 91.4-95.6), and 73.9% (95% CI, 71.6-76.3) and 78.7% (95% CI, 75.5-82.0), 86.0% (95% CI, 82.1-89.5), and 81.5% (95% CI, 78.9-83.7), respectively. The diagnostic performance of the CVS (AUC, 0.89 [95% CI, 0.86-0.91]) was superior to that of CLs (AUC, 0.77 [95% CI, 0.75-0.80]; *P* < .001), and was increased when combining the 2 imaging markers (AUC, 0.92 [95% CI, 0.90-0.94]; *P* = .04); in the random forest model, both CVS and CLs outperformed the presence of infratentorial, periventricular, and juxtacortical WMLs in supporting MS differential diagnosis.

**Conclusions and Relevance:**

The findings in this study suggest that CVS and CLs may be valuable tools to increase the accuracy of MS diagnosis.

## Key Points

**Question:**

Can multiple sclerosis (MS) be differentiated from a wide range of non-MS conditions showing brain white matter lesions using solely imaging biomarkers for cortical lesions (CLs) and central vein sign (CVS)?

**Findings:**

In this cross-sectional study including 1051 participants, the presence of CLs had high specificity and low sensitivity, while application of the 40% CVS rule resulted in high specificity and moderate sensitivity for MS diagnosis. CVS and CLs outperformed the contribution of infratentorial, periventricular, and juxtacortical lesions in supporting the diagnosis of MS.

**Meaning:**

The findings indicate that CVS and CLs may be valuable tools to increase the accuracy of MS diagnosis.

## Teaser

This cross-sectional study evaluates the use of cortical lesions and central vein sign in distinguishing multiple sclerosis from other conditions.

## Introduction

A wide range of conditions mimic the clinical presentation and the radiological features typical for multiple sclerosis (MS). The 2017 revised McDonald criteria^1^ have increased the sensitivity and shortened the time to diagnosis of MS,^2,3^ allowing earlier initiation of disease-modifying treatments. However, this came at the cost of a reduction in specificity compared to previous criteria,^2,3^ with misdiagnosis representing a relevant risk in clinical practice.^4,5,6^ Additionally, the McDonald criteria primarily apply to patients with typical demyelinating syndromes suggestive of MS,^1^ rendering the diagnostic workup of patients with atypical features challenging.

Cortical lesions (CLs) and the central vein sign (CVS) are magnetic resonance imaging (MRI) markers that have the potential to increase the accuracy of MS differential diagnosis.^7,8^ CLs are common in MS and typically absent or less prevalent in several MS-mimicking conditions.^7^ Despite the fact that only a limited proportion of CLs can be detected in vivo in patients with MS,^9,10^ the visualization of CLs can be significantly improved by using dedicated 3-dimensional (3-D) MRI sequences, including magnetization-prepared rapid gradient echo,^11^ magnetization-prepared 2 rapid acquisition gradient echo (MP2RAGE),^12^ double inversion recovery (DIR),^13^ and phase-sensitive inversion recovery (PSIR).^14^ Evidence that CLs increase the specificity of a diagnosis of MS in patients with clinically isolated syndrome (CIS)^15,16^ has led to the integration of CLs with juxtacortical lesions as an additional feature to demonstrate dissemination in space in the 2017 McDonald criteria.^1^

The CVS is a radiological finding detectable on susceptibility-based images, which consists of the presence of a vein in the center of a white matter lesion (WML). Due to the predominant perivenular distribution of MS plaques, the CVS is frequent in patients with MS^8^; conversely, lower prevalence has been consistently reported in several non-MS conditions, supporting the value of the CVS as a diagnostic biomarker.^8,17,18^

Currently, the role of the CVS and CLs in clinical practice is limited. The CVS is not integrated in the 2017 McDonald criteria and is not recommended for routine clinical use^19^; likewise, the acquisition of optimized MRI sequences for CL detection is only optional in the recently proposed international guidelines.^19^ This is mainly motivated by the insufficient evidence for a real advantage of these imaging biomarkers as compared to currently available ones and by the lack of widespread technology availability.^19^

This work aimed to characterize the value of CLs, the CVS, and their combination in differentiating MS from a wide spectrum of non-MS conditions presenting with WMLs in a large multicenter cohort. Additionally, we aimed to quantify the relative importance of CLs and CVS compared to conventional MRI biomarkers (ie, presence of periventricular, juxtacortical, and infratentorial WMLs) in supporting MS differential diagnosis.

## Methods

Written informed consent was obtained from all participants in each respective center. The study was approved by institutional review boards at each site. The data used in this study were shared in accordance with a MAGNIMS data transfer agreement. This study followed the Strengthening the Reporting of Observational Studies in Epidemiology (STROBE) reporting guideline.

### Participants

In this retrospective, cross-sectional study, we collected participants’ demographic, clinical, and MRI data from 14 European academic centers within the Magnetic Resonance Imaging in MS (MAGNIMS) framework and from a multicenter pan-European cohort (the European Prevention of Alzheimer’s Dementia cohort).^20^

Participants were eligible for inclusion if they (1) were older than 18 years; (2) had a brain 3-T MRI scan including fluid-attenuated inversion recovery/T2-weighted images, at least one 3-D sequence for CL assessment (among 3-D-T1, MP2RAGE, DIR, and PSIR), and 1 sequence for CVS detection (either 3-D T2*-weighted or susceptibility-weighted imaging [SWI]); (3) had WMLs; and (4) had a diagnosis of CIS,^1,21^ MS,^1,21^ or non-MS conditions. Non-MS conditions included myelin oligodendrocyte glycoprotein antibody-associated disease (MOGAD; defined as a demyelinating disorder in patients with positive myelin oligodendrocyte glycoprotein-IgG antibody test); aquaporin-4 (AQP4) IgG antibody–positive neuromyelitis optica spectrum disorder (NMOSD) and seronegative NMOSD diagnosed according to Wingerchuk 2015 criteria^22^; inflammatory vasculopathies; cerebrovascular disease; genetically confirmed Fabry disease; migraine with and without aura; and nonspecific WMLs in healthy control individuals. Patients were excluded from the study in case of clinical history positive for other neurological or psychiatric disorders. Whenever available, information regarding the presence of cerebrospinal fluid–restricted oligoclonal bands (OCBs) was systematically collected.

### MRI Analysis

The MRI acquisition protocol is detailed in eTable 1 in Supplement 1. CLs were defined as regions of (1) hyperintense signal on DIR or hypointense signal on 3-D-T1, MP2RAGE, or PSIR compared to the surrounding normal-appearing cortex; (2) extending for at least 3 mm along the main in-plane axis; and (3) partially or entirely involving the cortex.^7^ The presence of CLs was assessed independently by 2 raters (A.C. and R.C.) blinded to the participants’ diagnosis. In case of initial disagreement, consensus was achieved in a separate session. For participants who had multiple MRI sequences for CL detection available, the evaluation was performed independently for each contrast, on different sessions.

On 3-D-T1/MP2RAGE images, CLs were manually segmented and classified according to their location as intracortical or leukocortical. CL masks were used to create a CL probability map in patients with MS/CIS.^23^

The presence of a central vein was assessed on susceptibility-based images for all nonconfluent WMLs extending for at least 3 mm in the shortest diameter. The evaluation was performed by consensus of 2 raters (A.C. and R.C.) blinded to the participants’ identity, following North American Imaging in MS Cooperative (NAIMS) criteria.^8^ To estimate interrater agreement, a subset of 50 randomly selected participants was evaluated by the raters independently.

A second CL and CVS assessment was performed after blinding the raters to the general appearance of the scan (which could otherwise bias toward a specific diagnosis). CL assessment was repeated in a pool of 200 randomly selected images distributed among various MRI contrasts and participating centers to replicate the overall cohort’s distribution. CVS assessment was repeated on 1 randomly selected WML for each study participant. Further methodological details are available in the eMethods in Supplement 1.

### Statistical Analysis

Statistical analyses were conducted in R version 4.2.1 (R Foundation). We investigated the following.

- The performance of (1) CL count, (2) the proportion of CVS-positive lesions, and (3) their combination in discriminating between MS/CIS and non-MS conditions. Sensitivity, specificity, and accuracy for various CLs and CVS cutoffs were calculated. The combination of CLs and CVS was explored with a multivariable logistic regression model. Receiver operating characteristic (ROC) curves were obtained and compared using the DeLong method.^24^ Sensitivity analyses were conducted restricting the analysis to MS/CIS patients with less than 2 years of disease duration (eFigure 1 and eTable 2 in Supplement 1) and exploring the diagnostic accuracy of the CVS in the subgroup of patients with at least 3 lesions suitable for CVS assessment (eFigure 2 in Supplement 1). The diagnostic accuracy of the CVS was also assessed using the absolute number of CVS-positive lesions as cutoff (eFigure 3, eTable 3 in Supplement 1).
- Differences in CL burden and in the proportion of CVS-positive lesions between diagnosis groups (using negative binomial regression models and Mann-Whitney *U* test, respectively). Additionally, we explored the associations of CL count and the proportion of CVS-positive lesions with demographic and clinical variables in patients with MS (using negative binomial regression and logistic regression models, respectively).
- The relative importance of CLs, the CVS, and periventricular, juxtacortical, and infratentorial WMLs in differentiating between MS/CIS and non-MS conditions. The analysis was conducted by fitting a random forest model using the randomForest R package,^25^ and implemented using 500 trees (mtry = 2). For validation, data were randomly partitioned in training (two-thirds) and test (one-third) subsets, ensuring balanced distribution of MRI centers across groups. Variable importance was ranked by assessing the mean decrease in accuracy (MDA), computed from permutation of out-of-bag data.

Additionally, we explored the agreement (1) between raters in CL and CVS assessments and (2) in the CL count obtained on different MRI contrasts, using the intraclass correlation coefficient (ICC).^26^ We investigated the influence of the different susceptibility-weighted sequences on CVS diagnostic performance (eMethods in Supplement 1). We assessed the diagnostic performance of the CVS using a range of simplified criteria, alternative to the proportion of CVS-positive lesions, including the “Select-3,” “Pick-6,” and “Select-n*” algorithms.^27,28,29,30^ Such criteria have been proposed to expedite the evaluation process and promote the applicability of the CVS in clinical settings (eMethods, eFigures 10-12, and eTables 5-7 in Supplement 1). An estimation of the time required for conducting CL and CVS assessments is reported in the eMethods in Supplement 1.

In the subgroup of participants with data on OCBs status, we explored the relative value of OCBs, CLs, and CVS in differentiating between MS/CIS and non-MS conditions (eMethods in Supplement 1). Finally, post hoc analyses were conducted to further characterize the observed between-sex difference in CVS prevalence (eMethods in Supplement 1). The threshold of statistical significance was set at *P* < .05. Additional information regarding the statistical analysis is available in the eMethods in Supplement 1.

## Results

In total, 1051 participants were enrolled in the study with either MS/CIS (n = 599; 386 [64.4%] female; mean [SD] age, 41.5 [12.3] years) or non-MS conditions (including other neuroinflammatory disorders, cerebrovascular disease, migraine, and incidental WMLs in healthy control individuals; n = 452; 302 [66.8% female]; mean [SD] age, 49.2 [14.5] years). The cohort’s characteristics are summarized in the Table.

**Table.** Demographic and Clinical Characteristics

	MS (n = 550)	CIS (n = 49)	AQP4-positive NMOSD (n = 19)	Seronegative-NMOSD (n = 22)	MOGAD (n = 17)	Migraine (n = 96)	Inflammatory vasculopathies (n = 29)	Cerebrovascular disease (n = 63)	Fabry disease (n = 57)	Healthy control individuals (n = 149)
Male, No. (%)	198 (36)	15 (31)	3 (16)	3 (14)	11 (65)	20 (21)	10 (34)	29 (46)	26 (46)	48 (32)
Female, No. (%)	352 (64)	34 (69)	16 (84)	19 (86)	6 (35)	76 (79)	19 (66)	34 (54)	31 (54)	101 (68)
Age, mean (SD), y	41.9 (12.5)	37.2 (9.8)	47.3 (14.1)	47.3 (13.9)	39.8 (11.7)	42.3 (11.9)	51.9 (15.7)	63.2 (13.3)	42.3 (12.5)	51.4 (12.4)
Disease duration, median (IQR), y	6.0 (1.5-13.5)	0.4 (0.3-0.9)	3.6 (1.9-5.0)	7.0 (2.0-11.0)	4.3 (0.6-7.8)	NA	NA	NA	NA	NA
EDSS score, median (IQR)	2.0 (1.5-3.5)	2.0 (1.0-2.5)	3.25 (2.0-6.0)	3.0 (2.0-5.0)	1.5 (1.0-2.0)	NA	NA	NA	NA	NA
Disease subgroups, No. (%)	RRMS: 462 (84); SPMS: 62 (11); PPMS: 26 (5)	NA	NA	NA	NA	NA	Sjögren syndrome: 19 (66); SLE: 4 (14); Behçet disease: 3 (10); AAV: 2 (7); Susac syndrome: 1 (3)	CSVD: 53 (84); PFO: 10 (16)	NA	NA

Abbreviations: AAV, anti-neutrophil cytoplasmic antibody-associated vasculitis; AQP4, aquaporin-4 antibody; CIS, clinically isolated syndrome; CSVD, cerebral small vessel disease; MOGAD, myelin oligodendrocyte glycoprotein antibody-associated disease; MS, multiple sclerosis; NA, not applicable; NMOSD, neuromyelitis optica spectrum disorder; PFO, patent foramen ovale; PPMS, primary progressive multiple sclerosis; RRMS, relapsing-remitting multiple sclerosis; SLE, systemic lupus erythematosus; SPMS, secondary progressive multiple sclerosis.

For CL analysis, either 3-D-T1 (n = 722) or MP2RAGE (n = 329) images were available for all participants; DIR was available in 299 and PSIR in 20 participants. For CVS analysis, T2*-weighted images were available for 440 participants, while SWI was available for the remaining 611.

Three participants were excluded from CL analysis and 17 from CVS analysis due to insufficient MRI image quality. Five were excluded from the study due to missing clinical or demographic information (n = 3) or unclear diagnosis (n = 2).

### CLs

#### Discrimination Between MS/CIS and Non-MS Conditions

CLs were detected in 328 patients with MS (59.9%), 24 with CIS (49.0%), 1 with AQP4-positive NMOSD (5.3%), 3 with seronegative-NMOSD (13.6%), 4 with MOGAD (23.5%), 1 with migraine (1.1%), 7 with inflammatory vasculopathies (24.1%), 9 with cerebrovascular disease (14.3%), 0 with Fabry disease, and 4 healthy control individuals (2.7%). Examples are provided in eFigures 4 and 5 in Supplement 1. CL count was significantly higher in patients with MS/CIS compared to each non-MS condition.

**Figure 1.**
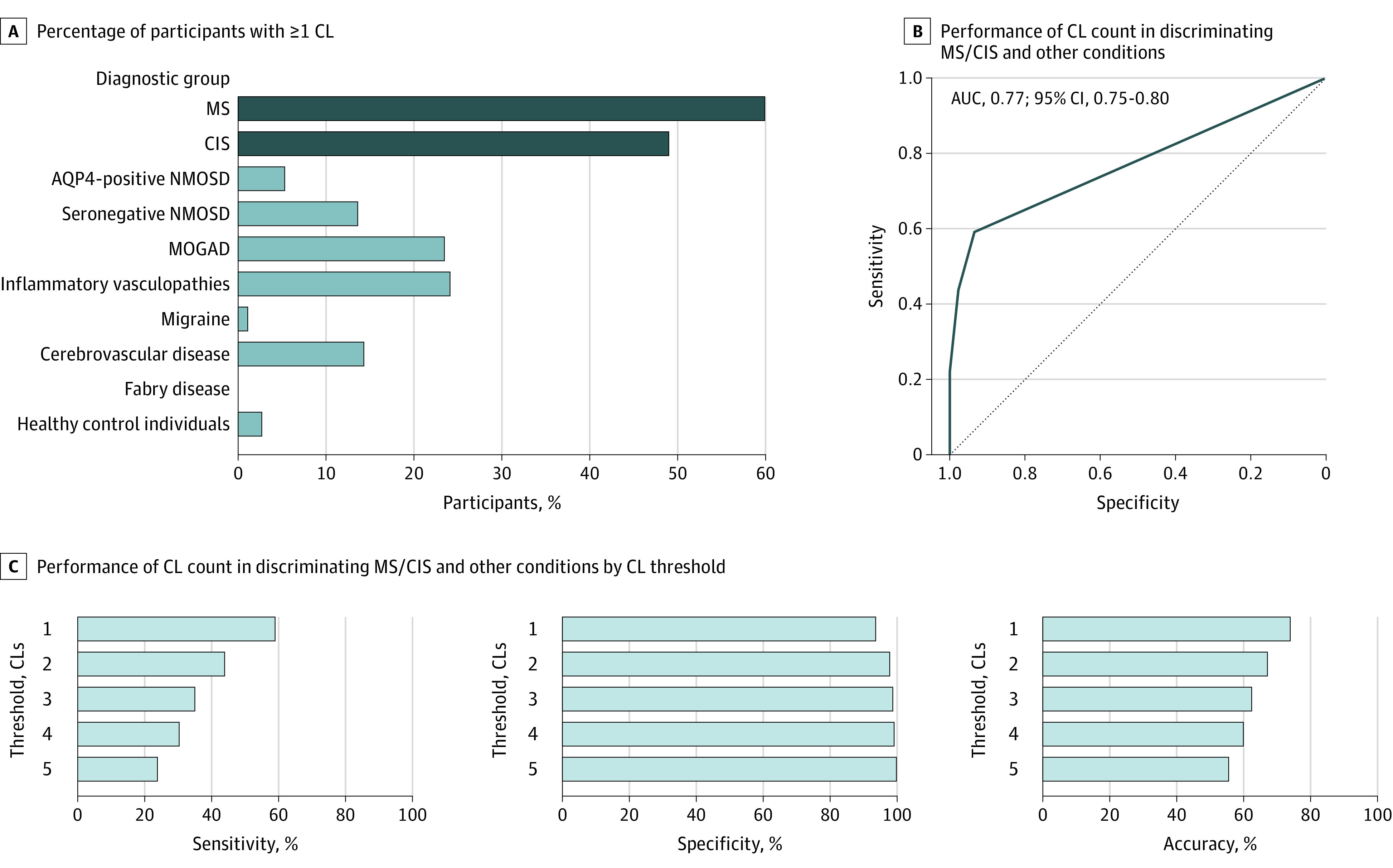
Cortical Lesions (CLs) for Discrimination Between Multiple Sclerosis (MS)/Clinically Isolated Syndrome (CIS) and Other Diagnoses AQP4 indicates aquaporin-4 antibody; AUC, area under the receiver operating characteristic curve; MOGAD, myelin oligodendrocyte glycoprotein antibody-associated disease; NMOSD, neuromyelitis optica spectrum disorder.

The ROC curve based on CL count had an area under the curve (AUC) of 0.77 (95% CI, 0.75-0.80). A cutoff of 1 CL achieved the highest discriminative performance (59.0% sensitivity [95% CI, 55.1-62.8], 93.6% specificity [95% CI, 91.4-95.6], and 73.9% accuracy [95% CI, 71.6-76.3] for a diagnosis of MS) (Figure 1).

**Figure 2.**
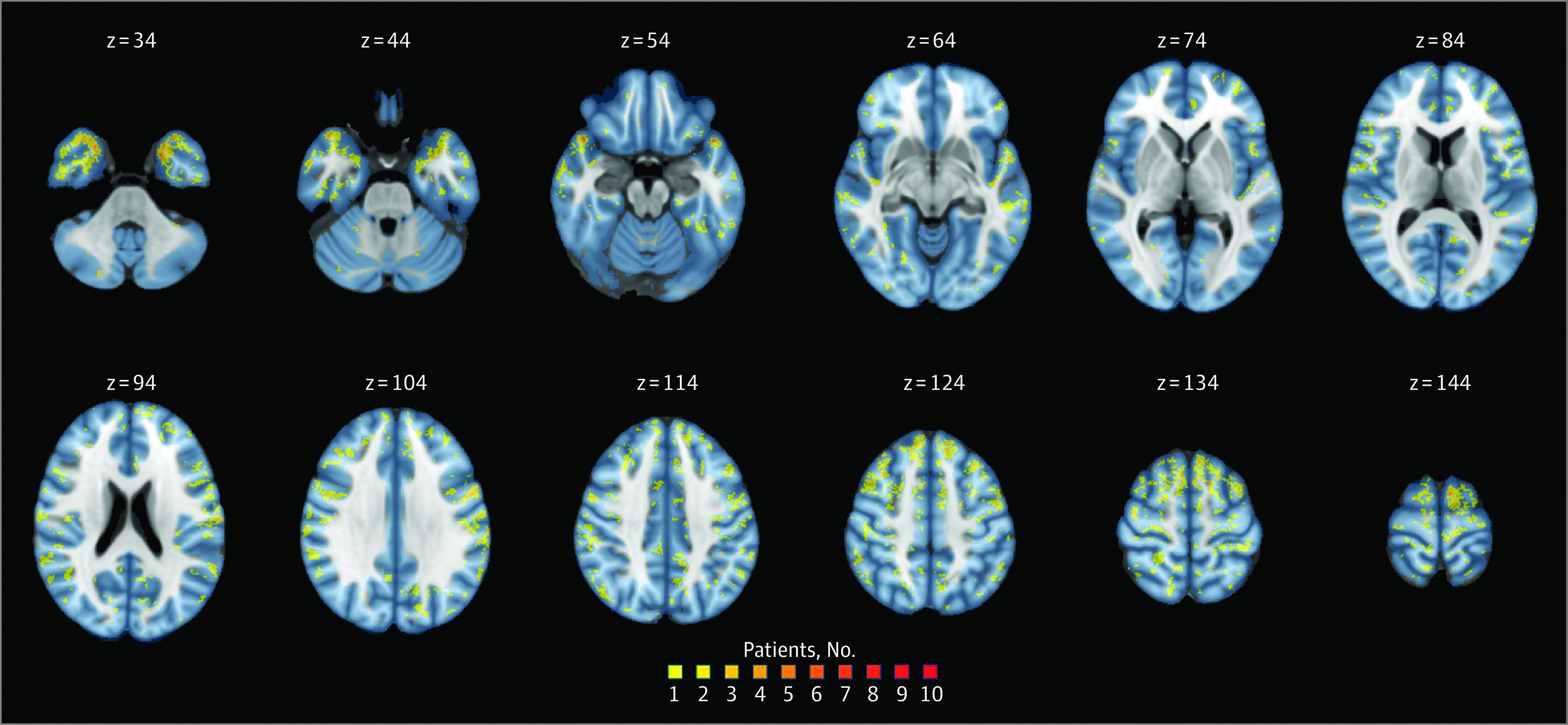
Cortical Lesion Probability Map in Patients With Multiple Sclerosis (MS)/Clinically Isolated Syndrome (CIS) The number of patients with MS/CIS presenting lesions in each cortical voxel is displayed in different colors, according to the reported color heat map. z indicates the z-axis coordinate of the Montreal Neurological Institute (MNI) 152 space.

CLs in patients with MS/CIS were most frequently involving the frontal lobe (321 of 597 patients [53.8%]), followed by parietal (204 of 597 patients [34.2%]) and temporal lobes (202 of 597 patients [33.8%]) (Figure 2). The distribution of CLs across brain lobes was not different between MS/CIS and non-MS conditions. Most CLs were leukocortical in both MS/CIS and non-MS groups (eTable 4 in Supplement 1).

#### CLs in Patients With MS

CL count in patients with MS was positively associated with age (incidence rate ratio [IRR], 1.03 [95% CI, 1.02-1.05]; *P* < .001), male sex (IRR, 1.41 [95% CI, 1.04-1.93]; *P* = .03), disease duration (IRR, 1.03 [95% CI, 1.01-1.05]; *P* < .001), and the Expanded Disability Status Scale score (IRR, 1.35 [95% CI, 1.24-1.47]; *P* < .001). Compared to patients with relapsing-remitting MS, patients with secondary progressive MS and primary progressive MS had higher CL burden (IRR, 2.47 [95% CI, 1.61-3.96]; *P* < .001 and IRR, 2.19 [95% CI, 1.18-4.60]; *P* = .02, respectively).

#### Additional Analyses

Excellent agreement in CL count was found between DIR and both 3-D-T1 (ICC, 0.95 [95% CI, 0.94-0.97]; n = 209) and MP2RAGE (ICC, 0.97 [95% CI, 0.96-0.98]; n = 90), and between PSIR and 3-D-T1 (ICC, 0.97 [95% CI, 0.93-0.99]; n = 20). Substantial interrater agreement in CL count was measured for all MRI contrasts considered (eMethods in Supplement 1).

### CVS

#### Discrimination Between MS/CIS and Non-MS Conditions

In total, 12 362 WMLs from 934 participants were analyzed (100 participants did not have WMLs suitable for CVS assessment according to NAIMS criteria).^8^ The median (IQR) proportion of CVS-positive lesions per patients was 62.1% (44.4-79.2) in MS, 68.4% (32.9-90.2) in CIS, 10.7% (0-40.5) in AQP4-positive NMOSD, 20.0% (0.0-50.0) in seronegative-NMOSD, 33.3% (13.7-50.0) in MOGAD, 0.95% (0.0-18.2) in migraine, 11.4% (0.0-30.1) in inflammatory vasculopathies, 0% (0.0-10.5) in cerebrovascular disease, 0% (0.0-1.4) in Fabry disease, and 0% (0.0-19.6) in healthy control individuals. Examples of CVS-positive and -negative lesions are provided in eFigure 6 in Supplement 1. The proportion of CVS-positive lesions per patient was significantly higher in MS/CIS compared to each non-MS condition.

**Figure 3.**
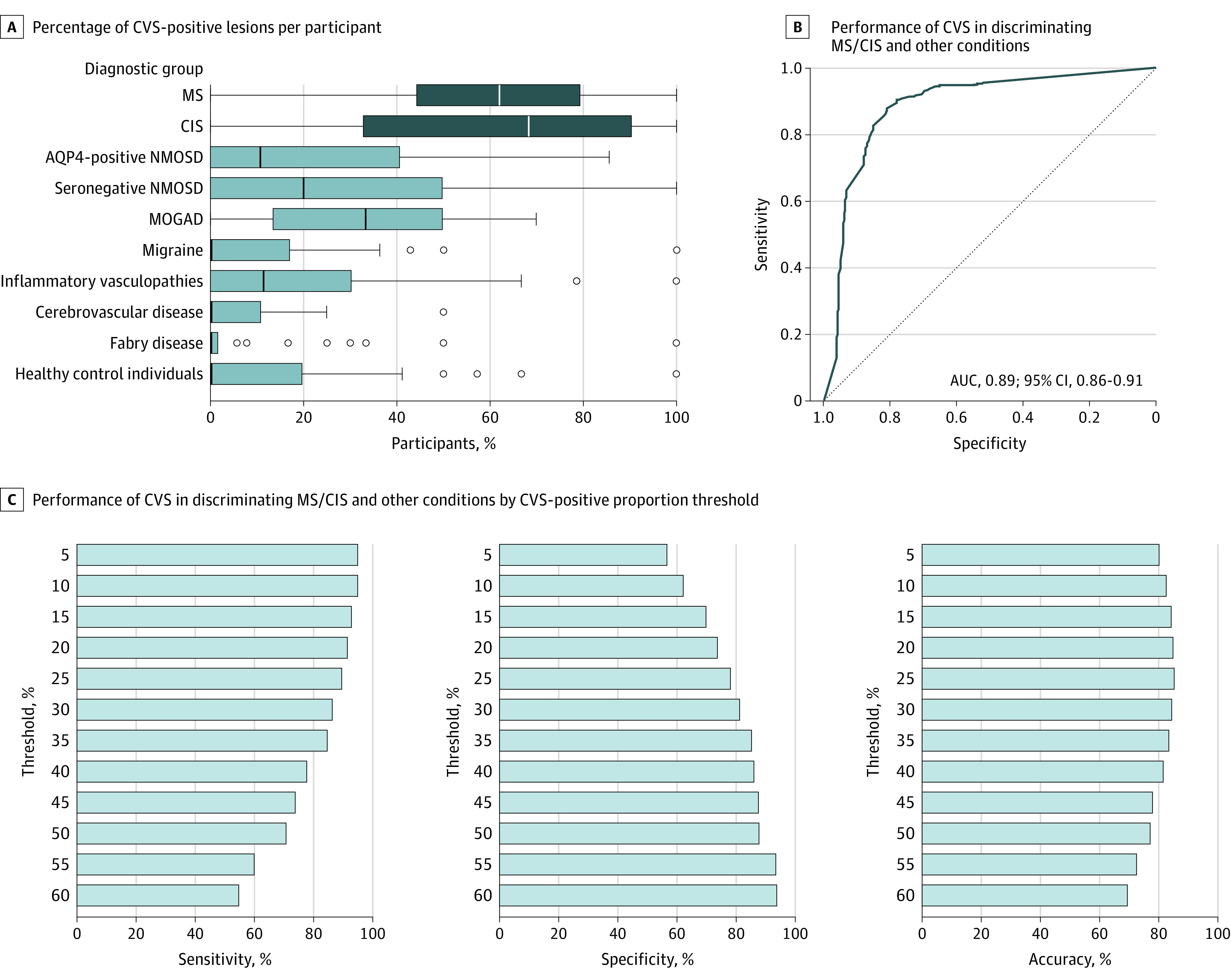
Central Vein Sign (CVS) for Discrimination Between Multiple Sclerosis (MS)/Clinically Isolated Syndrome (CIS) and Other Diagnoses AQP4 indicates aquaporin-4 antibody; AUC, area under the receiver operating characteristic curve; MOGAD, myelin oligodendrocyte glycoprotein antibody-associated disease; NMOSD, neuromyelitis optica spectrum disorder.

The ROC curve based on the proportion of CVS-positive lesions had an AUC of 0.89 (95% CI, 0.86-0.91) (Figure 3). The diagnostic performance was superior to that of CLs (AUC, 0.77 [95% CI, 0.75-0.80]; *P* < .001). Using the previously proposed 40% threshold,^27^ the CVS provided sensitivity, specificity, and accuracy of 78.7% [95% CI, 75.5-82.0], 86.0% [95% CI, 82.1-89.5], and 81.5% [95% CI, 78.9-83.7], respectively. Based on the Youden index, a 26% CVS-positive proportion threshold had the best discriminative performance (sensitivity, specificity, and accuracy: 88.0% [95% CI, 85.3-90.7], 80.9% [95% CI, 76.6-84.9], and 85.3% [95% CI, 83.0-87.6], respectively).

The proportion of CVS-positive lesions per topographical location, in both MS/CIS and non-MS, is reported in eFigure 7 in Supplement 1. The highest ratio in the proportion of CVS-positive lesions between MS/CIS and non-MS was measured in deep WMLs (5.9) (eFigure 8 in Supplement 1).

#### CVS in Patients With MS

The proportion of CVS-positive lesions in patients with MS was negatively associated with age (odds ratio [OR], 0.99 [95% CI, 0.99-0.99]; *P* < .001), female sex (OR, 0.58 [95% CI, 0.53-0.64]; *P* < .001), and disease duration (OR, 0.99 [95% CI, 0.99-1.00]; *P* = .02), while it was not associated with Expanded Disability Status Scale or disease course. Between-sex difference in the prevalence of CVS-positive lesions did not result in significant difference in CVS diagnostic performance between female and male participants (eMethods in Supplement 1).

#### Additional Analyses

The AUC based on the proportion of CVS-positive lesions was increased to 0.92 (95% CI, 0.90-0.94) when restricting the analysis to patients with at least 3 lesions suitable for CVS assessment (n = 773; best threshold for discrimination, 35%; sensitivity, 90.0% [95% CI, 87.4-92.5]; specificity, 81.7% [95% CI, 76.8-86.3]; accuracy, 87.5% [95% CI, 85.0-89.8]) (eFigure 2 in Supplement 1). The diagnostic performance of the CVS was not different when using either the proportion or the absolute number of CVS-positive lesions as the cutoff (AUC, 0.89 [95% CI, 0.86-0.91] vs 0.89 [95% CI, 0.87-0.91]; *P* = .45) (eFigure 3 and eTable 3 in Supplement 1).

The ICC for interrater agreement in CVS assessment was 0.95 (95% CI, 0.92-0.97). No difference in the diagnostic performance of the CVS was observed between T2*-weighted and SWI images (AUC, 0.89 [95% CI, 0.85-0.94] vs 0.87 [95% CI, 0.84-0.91], respectively; *P* = .45). The proportion of CVS-positive lesions in participants with MS/CIS was significantly higher on optimized submillimetric 3-D-EPI T2* images compared to SWI images (OR, 1.18 [95% CI, 1.08-1.30]; *P* < .001) (eMethods in Supplement 1).

Compared to the diagnostic performance achieved using the proportion of CVS-positive lesions, the performance of the simplified “Select-3” algorithm was reduced (AUC, 0.79 [95% CI, 0.75-0.84] vs 0.92 [95% CI, 0.89-0.95]; *P* < .001), while the performance of the “Select-6” and “Select-n*” algorithms was not different (AUC, 0.89 [95% CI, 0.85-0.93] vs 0.92 [95% CI, 0.87-0.96]; *P* = .23 and AUC, 0.86 [95% CI, 0.81-0.90] vs 0.88 [95% CI, 0.82-0.94]; *P* = .54, respectively). Classifying MS based on the presence of a minimum of 6 CVS-positive lesions, or in cases where there were fewer than 6 CVS-positive lesions on the prevalence of CVS-positive over CVS-negative lesions, yielded a sensitivity, specificity, and accuracy of 82.7% (95% CI, 77.8-87.5), 87.5% (95% CI, 78.6-94.7), and 83.6% (95% CI, 79.3-87.8), respectively (eMethods, eTables 5-7, and eFigures 10-12 in Supplement 1).

### Combination of CLs and CVS

**Figure 4.**
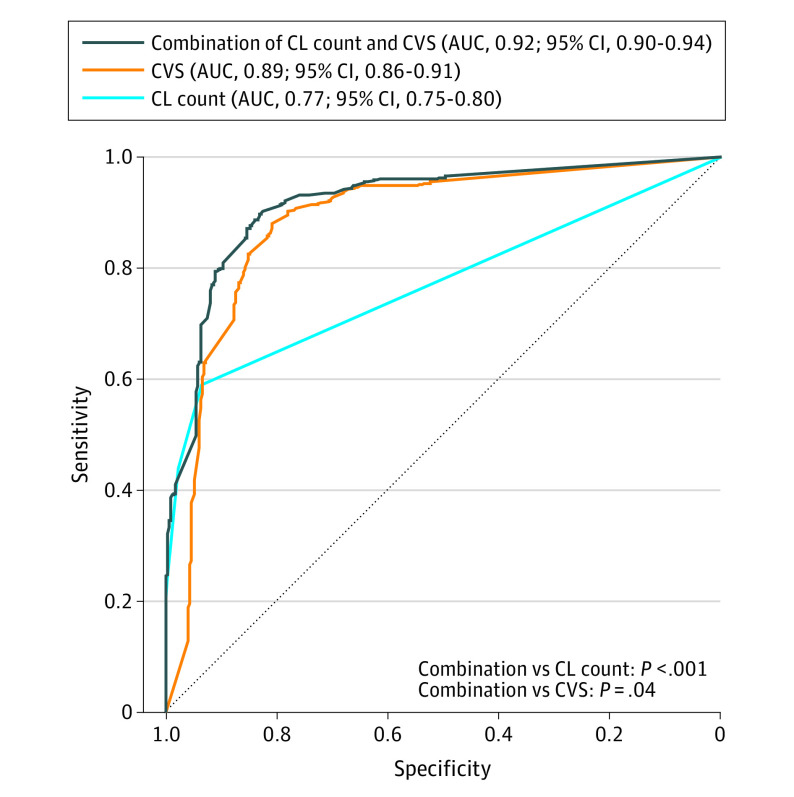
Combination of Cortical Lesions (CLs) and Central Vein Sign (CVS) for Discrimination Between Multiple Sclerosis/Clinically Isolated Syndrome and Other Diagnoses AUC indicates area under the receiver operating characteristic curve.

The diagnostic performance of the combination of CLs and CVS (AUC, 0.92 [95% CI, 0.90-0.94]) was higher compared to the performance of CLs (AUC, 0.77 [95% CI, 0.75-0.80]; *P* < .001) and CVS (AUC, 0.89 [95% CI, 0.86-0.91]; *P* = .04) alone (Figure 4). Within the subset of participants for whom OCBs data were available, the status of OCBs (OR, 17.8 [95% CI, 8.9-37.6]; *P* < .001), CLs (OR, 1.5 [95% CI, 1.2-2.2]; *P* = .005), and CVS (OR, 37.0 [95% CI, 11.6-133.3]; *P* < .001) each made significant and independent contributions to distinguishing between MS/CIS and non-MS conditions (eMethods in Supplement 1).

### CLs and CVS in Patients With Short Disease Duration

When the MS/CIS group was restricted to patients with less than 2 years of disease duration (n = 206, key clinical characteristics reported in eTable 4 in Supplement 1), the diagnostic performance of CLs was reduced compared to the performance in the entire cohort (AUC, 0.73 [95% CI, 0.69-0.76]; vs 0.77 [95% CI, 0.75-0.80]; *P* = .04), while the diagnostic performance of both CVS (AUC, 0.88 [95% CI, 0.85-0.91] vs 0.89 [95% CI, 0.86-0.91]; *P* = .75), and the combination of CLs and CVS (AUC, 0.90 [95% CI, 0.87-0.93] vs 0.92 [95% CI, 0.90-0.94]; *P* = .30) remained unaltered (eFigure 1 in Supplement 1).

### Random Forest

The AUC of the random forest model combining information on CLs, CVS, and WML location was 0.96 (95% CI, 0.94-0.97) in the training and 0.94 (95% CI, 0.91-0.97) in the test subset. In the ranking of variable importance, the CVS had the highest classification power (MDA, 57.2), followed by CLs (MDA, 37.8), presence of infratentorial (MDA, 15.0), periventricular (MDA, 13.2), and juxtacortical WMLs (MDA, 6.9).

## Discussion

In this large multicenter cross-sectional study, we quantified the performance of CLs, CVS, and their combination in differentiating MS/CIS from a wide range of non-MS conditions. The presence of CLs provided high specificity but low sensitivity, while the 40% CVS rule yielded high specificity and moderate sensitivity; the best performance was achieved by combining CL and CVS information. In a random forest model for discrimination between MS/CIS and non-MS conditions, the CVS exhibited the highest classification importance, followed by CLs; these 2 biomarkers outperformed the presence of WMLs in brain locations characteristic of MS (infratentorial, periventricular, and juxtacortical). Among a subgroup of participants with available OCB status, both CLs and CVS were shown to offer additional diagnostic value.

In line with previous evidence,^23^ we found CLs in most patients with MS, with increased prevalence in progressive phenotypes. As expected, however, the detection rate was inferior compared to the prevalence reported in pathological and 7T MRI studies.^9,10,31,32^ Accordingly, in our study, CLs provided only low sensitivity for the identification of patients with MS/CIS. Conversely, the presence of a single CL provided a specificity for MS/CIS diagnosis of nearly 94%.

Notably, the detection rate of CLs was similar between 3-D-T1/MP2RAGE and both DIR and PSIR. This evidence might have practical implications since 3-D T1-weighted images are increasingly available in clinical practice; moreover, the inclusion of 3-D T1-weighted images in the MRI protocol of patients with MS would also be beneficial since it allows the quantitative assessment of brain volumes.^19^

A higher proportion of CVS-positive lesions was found in patients with MS/CIS compared to all the other conditions considered, resulting in high diagnostic performance. High specificity and moderate sensitivity were reached using the previously proposed 40% cutoff.^27^ Interestingly, in our cohort a lower cutoff (26%) showed the highest discriminative performance based on the Youden index, confirming the results of a similar previous multicenter study.^33^ The differences in the optimal thresholds obtained in different studies are likely due to the heterogeneity of MRI acquisitions and image postprocessing techniques applied as well as the differential diagnoses included and the clinical and demographic characteristics of the participants enrolled. Notably, in our study, the optimal threshold was increased to 34% when considering exclusively the scans acquired with a submillimetric-resolution 3-D-EPI gradient-echo sequence; this evidence supports the concept that sequences more sensitive to the CVS allow the use of higher cutoffs (which increase specificity without excessive decrease in sensitivity).^33^ Interestingly, the optimal threshold also increased to 35% when excluding patients with less than 3 lesions suitable for CVS assessment, in whom the reliability of the CVS-proportion was likely affected by the low number of WMLs.

In agreement with previous investigations,^8,33,34^ the proportion of CVS-positive lesions was not different among MS phenotypes, was negatively associated with age, and was highest in periventricular lesions both in patients with MS and in non-MS conditions. Although the proportion of CVS-positive lesions was higher in male participants, this discrepancy did not result in significant between-sex differences in the diagnostic performance of the CVS. It might be speculated that female individuals exhibit decreased venule visibility on the susceptibility-based MRI because of overall lower iron levels. This aligns with the evidence that the between-sex differences were not MS-specific but were also evident in non-MS conditions. Future studies should better explore these differences.

The diagnostic performance of the CVS was superior to that of CLs. This finding was further supported by the random forest model, where the CVS had the highest importance in the discrimination between MS/CIS and non-MS conditions. When integrating CVS and CL information, the diagnostic performance of the CVS was increased, but only marginally. Additionally, differently from CLs, the diagnostic performance of the CVS was not reduced in patients with short disease duration—a subgroup of patients that is more representative of those undergoing diagnostic workups in a clinical setting. Overall, our data further support the value of the CVS to optimize the accuracy of MS diagnosis. Considering that MRI optimized for CVS can now be obtained with commercially available sequences and short acquisition times, our study supports the recently proposed inclusion of the CVS in the MS diagnostic criteria.^35^ In addition, the acquisition of susceptibility-based images would also be beneficial in patients with MS since it allows the identification of paramagnetic rim lesions, an emerging prognostic biomarker of chronic smoldering inflammatory activity.^36,37^

Remarkably, within our study population we also showed that simplified criteria for CVS assessment such as the “Select-n*” algorithm, which significantly expedite the evaluation process, can provide diagnostic accuracy comparable to the more time-consuming examination of all WMLs. This finding highlights the potential value of the CVS as a robust MRI biomarker even in clinical scenarios where time constraints play a critical role.

The assessment of the CVS was not possible in the 9.5% of participants included in our cohort due to the absence of lesions fulfilling the NAIMS criteria. In addition, the diagnostic value of the CVS in patients with only a few WMLs suitable for assessment was reduced. In this context, CLs—with their high positive predictive value—allow to reach a higher performance compared to the presence of WMLs in brain locations typical of MS and thus enhance diagnostic accuracy.

### Strengths and Limitations

This study has several strengths. First, it was conducted in a very large data sample, which was collected across multiple centers. It also encompassed a wide spectrum of conditions mimicking MS, and included a large cohort of patients with MS/CIS, which permitted a comprehensive exploration of the impact of clinical and demographic factors on the diagnostic performance of CLs and CVS. Second, to our knowledge, this is the first study performing the simultaneous evaluation of CLs and CVS as diagnostic MS biomarkers. Besides, it is a work where we also evaluated the comparative diagnostic performance of WMLs, and of OCBs status for a subgroup of participants. Third, the study compares the performance of different MRI protocols for CL and CVS assessment, along with various sets of simplified criteria for the CVS, hereby permitting to explore their clinical utility.

This study also has limitations. While the aim of the study was to estimate the diagnostic value of CLs and CVS in a real-world setting, the heterogeneity of the MRI data included may have constituted a bias due to the unequal distribution of the different diagnostic entities among the different MRI protocols. Similarly, the interpretation of the comparisons of CL count obtained on different MRI contrasts is complicated by the heterogeneity in MRI protocols (including different resolutions). The primary results of the study are based on a cohort of participants with heterogeneous disease duration. Nonetheless, the value of CLs and CVS was also explored in a large subgroup of participants with short disease duration, reflecting hereby a classical clinical diagnostic scenario. We assessed the diagnostic performance of CLs and CVS by considering the fulfillment of McDonald diagnostic criteria as the reference standard, acknowledging the inherent limitations of these criteria. This constraint, which motivates the research for biomarkers enhancing MS diagnosis accuracy, was likely mitigated in our cohort by the fact that all patients underwent evaluation in specialized academic centers with a high level of expertise in MS management. In addition, our study suffers from the intrinsic limitations of a cross-sectional design. Prospective studies would allow better characterization of the accuracy of diagnostic classification. Due to the lack of spinal cord MRI images, we were not able to compare the diagnostic performance of CLs and the CVS with the performance of the entire set of McDonald MRI criteria for dissemination in space.

## Conclusions

In MS differential diagnosis, the presence of CLs on 3T MRI images provided high specificity and low sensitivity while the 40% CVS rule yielded high specificity and moderate sensitivity. CVS and CLs outperformed the presence of infratentorial, periventricular, and juxtacortical WMLs in supporting the differentiation between MS/CIS and non-MS conditions. CVS and CLs, as assessed on dedicated MRI sequences, may be valuable tools to optimize the accuracy of MS diagnosis.

## Notes

Supplement 1. eMethods eTable 1. Overview of MRI protocols eTable 2. Comparison of the clinical, demographics, and MRI characteristics of the cohort with <2 years of disease duration and the entire cohort eFigure 1. Diagnostic performance of CLs, CVS, and their combination in the entire cohort and in the subgroup of patients with < 2 years of disease duration eFigure 2. Comparison of CVS diagnostic performance in the entire cohort vs in subjects with ≥3 lesions suitable for assessment eFigure 3. Comparison of CVS diagnostic performance: using a proportion-based threshold vs using the absolute number of CVS-positive lesions eTable 3: Sensitivity, specificity and accuracy for various absolute number of CVS-positive lesions eTable 4. Prevalence of cortical lesion subtypes among different diagnosis groups eFigure 4. Examples of cortical lesions in patients with multiple sclerosis eFigure 5. Examples of cortical lesions in non-MS conditions eFigure 6. Examples of images used for CVS assessment eFigure 7. Proportion of CVS-positive lesions per location in MS/CIS and non-MS conditions eFigure 8. Ratio of CVS-positive lesions between MS/CIS and non-MS conditions per location eFigure 9. Examples of images used to blind raters to the general appearance of the scan eTable 5. Diagnostic performance of the “Select-3” algorithm eFigure 10. Comparison of the diagnostic performance of the “Select-3” algorithm and the proportion of CVS-positive lesions eTable 6. Diagnostic performance of the “Pick-6” algorithm eFigure 11. Comparison of the diagnostic performance of the “Pick-6” algorithm and the proportion of CVS-positive lesions eTable 7. Diagnostic performance of the “Select-n*” algorithm eFigure 12. Comparison of the diagnostic performance of the “Select-n*” algorithm and the proportion of CVS-positive lesions eReferences

Supplement 2. Members of the Magnetic Resonance Imaging in MS (MAGNIMS) Study Group

Supplement 3. Data sharing statement
