# Supplementary material for: Diagnostic Performance of Cortical Lesions and the Central Vein Sign in Multiple Sclerosis
Source: JAMA Neurol. 2023 Dec 11;81(2):143–53. doi: 10.1001/jamaneurol.2023.4737 (PMC10714285; doi:10.1001/jamaneurol.2023.4737)
Supplement: Supplement 1. — eMethods eTable 1. Overview of MRI protocols eTable 2. Comparison of the clinical, demographics, and MRI characteristics of the cohort with <2 years of disease duration and the entire cohort eFigure 1. Diagnostic performance of CLs, CVS, and their combination in the entire cohort and in the subgroup of patients with < 2 years of disease duration eFigure 2. Comparison of CVS diagnostic performance in the entire cohort vs in subjects with ≥3 lesions suitable for assessment eFigure 3. Comparison of CVS diagnostic performance: using a proportion-based threshold vs using the absolute number of CVS-positive lesions eTable 3: Sensitivity, specificity and accuracy for various absolute number of CVS-positive lesions eTable 4. Prevalence of cortical lesion subtypes among different diagnosis groups eFigure 4. Examples of cortical lesions in patients with multiple sclerosis eFigure 5. Examples of cortical lesions in non-MS conditions eFigure 6. Examples of images used for CVS assessment eFigure 7. Proportion of CVS-positive lesions per location in MS/CIS and non-MS conditions eFigure 8. Ratio of CVS-positive lesions between MS/CIS and non-MS conditions per location eFigure 9. Examples of images used to blind raters to the general appearance of the scan eTable 5. Diagnostic performance of the “Select-3” algorithm eFigure 10. Comparison of the diagnostic performance of the “Select-3” algorithm and the proportion of CVS-positive lesions eTable 6. Diagnostic performance of the “Pick-6” algorithm eFigure 11. Comparison of the diagnostic performance of the “Pick-6” algorithm and the proportion of CVS-positive lesions eTable 7. Diagnostic performance of the “Select-n*” algorithm eFigure 12. Comparison of the diagnostic performance of the “Select-n*” algorithm and the proportion of CVS-positive lesions eReferences [file jamaneurol-e234737-s001.pdf]

Supplemental Online Content

Cagol A, Cortese R, Barakovic M, et al; MAGNIMS Study Group. Diagnostic performance of cortical lesions and the central vein sign in multiple sclerosis. *JAMA Neurol*. Published online December 11, 2023. doi:10.1001/jamaneurol.2023.4737

eMethods
eTable 1. Overview of MRI protocols
eTable 2. Comparison of the clinical, demographics, and MRI characteristics of the cohort with <2 years of disease duration and the entire cohort
eFigure 1. Diagnostic performance of CLs, CVS, and their combination in the entire cohort and in the subgroup of patients with < 2 years of disease duration
eFigure 2. Comparison of CVS diagnostic performance in the entire cohort vs in subjects with ≥3 lesions suitable for assessment
eFigure 3. Comparison of CVS diagnostic performance: using a proportion-based threshold vs using the absolute number of CVS-positive lesions
eTable 3: Sensitivity, specificity and accuracy for various absolute number of CVS-positive lesions
eTable 4. Prevalence of cortical lesion subtypes among different diagnosis groups
eFigure 4. Examples of cortical lesions in patients with multiple sclerosis
eFigure 5. Examples of cortical lesions in non-MS conditions
eFigure 6. Examples of images used for CVS assessment
eFigure 7. Proportion of CVS-positive lesions per location in MS/CIS and non-MS conditions
eFigure 8. Ratio of CVS-positive lesions between MS/CIS and non-MS conditions per location
eFigure 9. Examples of images used to blind raters to the general appearance of the scan
eTable 5. Diagnostic performance of the “Select-3” algorithm
eFigure 10. Comparison of the diagnostic performance of the “Select-3” algorithm and the proportion of CVS-positive lesions
eTable 6. Diagnostic performance of the “Pick-6” algorithm
eFigure 11. Comparison of the diagnostic performance of the “Pick-6” algorithm and the proportion of CVS-positive lesions
eTable 7. Diagnostic performance of the “Select-n*” algorithm
eFigure 12. Comparison of the diagnostic performance of the “Select-n*” algorithm and the proportion of CVS-positive lesions
eReferences

This supplemental material has been provided by the authors to give readers additional information about their work.

© 2023 Cagol A et al. *JAMA Neurology.*

eMethods

MRI analysis

T2-hyperintense white matter lesions (WMLs) were segmented using a deep-learning based tool.[1] The automatic outputs were checked, and manually edited when needed.
According to their location, WMLs were manually classified in periventricular (i.e. in direct contact with the lateral ventricles), juxtacortical (i.e. in direct contact with the cortex), infratentorial (involving the brainstem, cerebellum, or cerebellar peduncles), and deep white matter.[2]

Cortical lesions (CLs) and the central vein sign (CVS) were rated by two neurologists (A.C. and R.C.). CL assessment was performed following the MAGNIMS consensus recommendations for DIR images,[3] and following the criteria proposed by Sethi *et al* for PSIR images.[4]
In patients with availability of multiple MRI sequences suitable for CL detection, the evaluation was performed independently for each contrast, on different sessions separated in time by at least one month to minimize recall of lesion presence/location. Since DIR and PSIR images were available only for a subset of patients, with unbalanced distribution among different diagnosis groups, the CL count used in the statistical analyses was the count obtained on 3D-T1/MP2RAGE images.
CL masks were created by manually segmenting CLs on 3D-T1/MP2RAGE images, and used to generate a CL probability map in patients with MS/CIS.[5] Specifically, 3D-T1/MP2RAGE images of patients with MS/CIS having CLs were first brain-extracted using *HD-BET*,[6] and then non-linearly registered to the MNI152 template using *Advanced Neuroimaging Tools* (*ANTs*).[7] Registration results were visually checked to ensure accuracy. The obtained transformation matrices were then used to register CL masks to the MNI152 space, using the nearest neighbor interpolation. Topography of CLs was defined according to the Harvard-Oxford atlas (*https://fsl.fmrib.ox.ac.uk/fsl/fslwiki/Atlases*).

For CVS assessment, lesions smaller than 3 mm in the shortest diameter were automatically removed from the WML masks. The resulting masks were manually refined to remove confluent lesions, registered to the space of the susceptibility-based image, and used as reference for CVS rating. Whenever T2*-weighted and FLAIR images were available, the combined FLAIR* contrast was generated.[8]

Since CL and CVS assessments were performed on native images, in which the location and morphology of WMLs may bias the rater toward a specific diagnosis, additional analyses were performed to quantify the reproducibility of the assessments after blinding the raters to the general appearance of the scan.
For CLs, a subset of 200 randomly-selected 3D-T1/MP2RAGE images (distributed among various MRI contrasts and participating centers to replicate the overall cohort's distribution) were re-assessed for the presence of CLs after nullifying the white matter signal. Specifically, an automatic segmentation of the white matter was obtained with *SAMSEG*;[9] the segmentation was then used to remove the white matter signal from the native images (after a one-voxel erosion, to preserve the border between cortical gray matter and white matter in the final images; **eFigure 9, panel A**).
The agreement in CL count obtained on native images and on white matter-nullified images, as measured with the intraclass correlation coefficient (ICC),[10] was 0.96 (95% confidence interval [CI]: 0.94-0.97).
For CVS, one randomly-selected WML for each participant was re-assessed for the presence of CVS after being cropped from the native image (**eFigure 9, panel B**). The agreement in the CVS rating on native and cropped images, as measured with the Cohen's kappa coefficient, was 0.847 (95%-CI: 0.811-0.884).

CL subtypes

On native 3D-T1/MP2RAGE images, CLs were manually categorized – by consensus – in intracortical (i.e. confined to the cortex), and leukocortical (i.e. concomitantly involving the cortex and white matter). Leukocortical lesions were further differentiated in those with prevalent gray matter/white matter involvement. The presence of curvilinear/"worm-shaped" CLs[2] was also recorded. The prevalence of CL subtypes in the different diagnosis groups is reported in **eTable 4**.

Inter-rater agreement in CL count

The inter-rater agreement in CL count was estimated with the ICC, using a two-way mixed-effects model. The inter-rater agreement was estimated separately for each MRI contrast used for CL assessment.

- For 3D-T1 (n=720) the ICC for absolute inter-rater agreement was 0.97 (95%-CI: 0.97-0.98).
- For MP2RAGE (n=328) the ICC for absolute inter-rater agreement was 0.98 (95%-CI: 0.98-0.99).
- For PSIR (n=20) the ICC for absolute inter-rater agreement was 0.92 (95%-CI: 0.81-0.97).

© 2023 Cagol A et al. *JAMA Neurology.*

- For DIR (n=299) the ICC for absolute inter-rater agreement was 0.80 (95%-CI: 0.74-0.84).

Comparison between sequences for CVS assessment

We compared the diagnostic performance of the CVS on different susceptibility-based sequences by using the DeLong method.[11] Additionally, we compared the proportion of CVS-positive lesions on different susceptibility-based sequences in patients with MS/CIS by using logistic regression models, adjusted for age, sex, and disease duration.

The diagnostic performance of the CVS was not significantly different in the subgroup of subjects with T2*-weighted images and in the subgroup of subject with SWI images (AUC=0.893 [95%-CI: 0.850-0.935] and AUC=0.872 [95%-CI: 0.839-0.905], respectively; p=.45). Similarly, there was no difference in the diagnostic performance in the subgroup of subjects with optimized, submillimetric 3D-EPI images and in the subgroup of subjects with SWI images (AUC=0.877 [95%-CI: 0.817-0.937] and AUC=0.872 [95%-CI: 0.839-0.905], respectively; p=.88). Based on Youden's index, the best threshold for discrimination between MS/CIS and non-MS was 26% on SWI images, and 34% on 3D-EPI images.

The proportion of CVS-positive lesions in patients with MS/CIS was not significantly different between T2*-weighted and SWI images (odds ratio [OR]: 1.024; p=.60), while it was significantly higher on optimized, submillimetric 3D-EPI images compared to SWI images (OR: 1.182; p<.001).

Random forest model

The random forest model was fitted in the group of patients with availability of both CL and CVS data (n=932). The variables included in the random forest model were: 1) the proportion of CVS-positive lesions, 2) CL count, 3) presence/absence of periventricular WMLs, 4) presence/absence of juxtacortical WMLs, and 5) presence/absence of infratentorial WMLs.

In a sensitivity analysis, CVS and CL were used as dichotomous variables: specifically, the fulfillment of the "40%-CVS rule" and the presence/absence of CL were the variables entered in the model, together with the presence/absence of periventricular, juxtacortical, and infratentorial WMLs. The AUC was 0.931 (95%-CI: 0.912; 0.951) in training, and 0.905 (95%-CI: 0.869-0.940) in test subsets. The mean decrease in accuracy (MDA) was 63.0 for CVS, 39.2 for CLs, 16.3 for infratentorial WMLs, 12.8 for periventricular WMLs, and 10.6 for juxtacortical WMLs.

Analyses within the subgroup of participants with availability of oligoclonal bands information

Information on CSF-specific oligoclonal bands (OCBs) status was available for 505 participants: 371 with MS, 48 with CIS, 14 with AQP4-positive NMOSD, 16 with seronegative-NMOSD, 13 with MOGAD, 8 with migraine, 25 with inflammatory vasculopathies, and 10 with cerebrovascular disease.

Within this cohort, the presence of CSF-specific oligoclonal bands was associated with a sensitivity of 87.1%, a specificity of 80.2%, and an accuracy of 85.9% for the discrimination between MS/CIS and non-MS.

We assessed the relative contribution of i) OCBs status, ii) CL count, and iii) the proportion of CVS-positive lesions in supporting the distinction between MS/CIS and non-MS conditions using a multivariable logistic regression model. Specifically, the diagnosis (MS/CIS vs non-MS) was used as the dependent variable, while OCBs status, CL count, and the proportion of CVS-positive lesions were included as independent variables.

In this model, all three biomarkers demonstrated significant and independent associations with the diagnosis, as outlined below:

Variable	OR (95%-CI)	Z-value	P-value
OCBs status	17.785 (8.900; 37.567)	7.875	**<0.0001**
CL count	1.533 (1.195; 2.161)	2.839	**0.005**
Proportion of CVS-positive lesions	37.0 (11.598; 133.277)	5.826	**<0.0001**
AUC: 0.942 (95%-CI: 0.915-0.969)			

Similar results were obtained in a model using CLs and CVS as dichotomous variables (defined by thresholds of 1 CL and 40% CVS-positive proportion, respectively):

Variable	OR (95%-CI)	Z-value	P-value
OCBs status	19.729 (10.067; 40.861)	8.387	**<0.0001**
≥1 CL	3.303 (1.582; 7.190)	3.114	**0.002**
≥40% CVS-positive proportion	7.765 (3.941; 15.867)	5.796	**<0.0001**
AUC : 0.928 (95%-CI: 0.897-0.959)			

© 2023 Cagol A et al. *JAMA Neurology.*

Between-sex differences in the proportion of CVS-positive lesions

Post-hoc analyses were conducted to further characterize the observed difference in CVS prevalence between sexes. The analyses encompassed:

1. the assessment of the association between the proportion of CVS-positive lesions and sex in participants with MS, adjusting for potential clinical, demographic, and MRI confounding factors. The analysis was conducted with a multivariable logistic regression model including age, disease duration, EDSS, disease course, and white matter lesion load as covariates;
2. the assessment of the association between the proportion of CVS-positive lesions and sex in participants with non-MS conditions. The analysis was conducted with a logistic regression model using sex as exploratory variable;
3. a comparison of the diagnostic performance of the CVS in females and males. The performance of the proportion of CVS-positive lesions in discriminating between MS/CIS and non-MS conditions was explored with ROC curves separately in males and females. The AUC of the ROC curves obtained in the two groups was compared with the DeLong method.[11]

The results confirmed a negative association between the proportion of CVS-positive lesions and female sex among participants with MS also when adjusting for relevant clinical, demographic, and MRI factors [OR: 0.611 (95%-CI: 0.554-0.674), p<0.0001; Variance Inflation Factor (VIF) for all the variables <1.5].

A significant negative association between the proportion of CVS-positive lesions and female sex was also observed in the non-MS group [OR: 0.588 (95%-CI: 0.478; 0.723), p<0.0001].

The performance of CVS in supporting the differentiation between MS/CIS and non-MS conditions was not significantly different between females and males [AUC in females: 0.877 (95%-CI: 0.845-0.908); AUC in males: 0.907 (95%-CI: 0.870-0.945); p=0.22].

Simplified criteria for CVS assessment

Due to the need to evaluate all lesions that meet the NAIMS criteria in order to estimate the proportion of CVS-positive lesions, this process can be time-consuming, potentially hindering its practical use in clinical settings. Consequently, various simplified criteria have been proposed in the literature. In our study, we have investigated the diagnostic performance of four simplified algorithms for CVS assessment in a subset of the study cohort:

1. "Select-3"[12-14]: for each patient, 3 lesions of the subcortical or deep white matter were randomly selected on FLAIR/T2 images. Scans with <3 candidate lesions were excluded. The selected lesions were then assessed for the presence of the CVS on the susceptibility-based contrast.
2. "Pick-6"[13-14]: for each patient, 6 white matter lesions were randomly selected on FLAIR/T2 images. Scans with <6 candidate lesions were excluded. The selected lesions were then assessed for the presence of the CVS on the susceptibility-based contrast.
3. "Select-n*"[12,14-16]: the presence of CVS-positive lesions was directly determined on the FLAIR* contrast. Participants were classified as having MS if at least n lesions displayed the CVS over the entire FLAIR* image, with n ranging from 1 ("Select-1*") to 6 ("Select-6*").
4. The performance of the "Select-6*" approach was also assessed with an algorithm ("Select-6*[2]") classifying participants as having MS if they met one of the two following criteria: 1. at least 6 lesions exhibiting the CVS; 2. CVS-positive lesions outnumbering CVS-negative lesions, in case fewer than 6 CVS-positive lesions were present.[14,15,17]

The performance of the "Select-3" and "Pick-6" algorithms was assessed in a subgroup consisting of half of the study cohort (ensuring that the selected group had a distribution across centers/MRI protocols and diagnoses representative of the entire cohort).

The performance of the "Select-n*" algorithms was assessed in all participants with the availability of a 3D-EPI contrast (n=310).

For all algorithms, we assessed the diagnostic performance for various thresholds, with ROC curves. We then compared the diagnostic performance of each algorithm to that of the proportion of CVS-positive lesions within the same subset of participants using the DeLong method.[11]

Results of the analyses are reported in eTables 7-5 and eFigures 10-12.

Average duration for CL and CVS assessments

© 2023 Cagol A et al. *JAMA Neurology.*

The time required to conduct CL and CVS assessments was quantified in a subgroup of participants. Specifically:

- For the CL assessment, we randomly selected 20 3D-T1 or MP2RAGE scans, ensuring a balanced distribution across MRI protocols and centers. Two raters independently evaluated the selected scans and measured the time needed to complete the assessment for each scan.
- For the CVS assessment, we randomly selected 20 scans. To account for the significant impact of white matter lesion load on CVS assessment time, we ensured that 5 scans were selected from each of the four quartiles of the distribution of the white matter lesion load within the study population. The scans were selected from participants who had T2* contrast availability, allowing us to compare the time required for assessing the proportion on CVS-positive lesions with that for CVS assessment based on the Select-6*[2] algorithm. As for the CL assessment, two raters independently evaluated the selected scans and measured the time needed to complete the assessment for each scan.

The median time required to complete the CL assessment was 4.3 minutes (range: 3.4-6.4). The median time required to complete the CVS assessment based on the proportion of CVS-positive lesions was 3.3 minutes (range: 1.0-8.5). The median time required to complete the CVS assessment based on the Select-6*[2] algorithm was 1.1 minutes (range: 0.9-1.6), which was significantly shorter compared to the time required for the assessment based on the proportion of CVS-positive lesions ($p<.001$).

© 2023 Cagol A et al. *JAMA Neurology.*

eTable 1. Overview of MRI protocols

		3D-T1	MP2RAGE	DIR	PSIR	T2/FLAIR	GRE sequence
**Amsterdam** (n=40)	*Type*	/	NA	NA	NA	3D-FLAIR	SWI
	Resolution (mm)	0.94x0.94x1.0				0.98x0.98x1.2	0.49x0.49x3.0
	n	40				40	40
**Barcelona #1** (n=43)	*Type*	/	NA	NA	NA	TIRM, tra	SWI
	Resolution (mm)	1.0x1.0x1.0				0.49x0.49x2.99	0.65x0.65x3.0
	n	43				43	43
**Barcelona #2** (n=16)	*Type*	/	NA	NA	NA	TIRM, tra	SWI
	Resolution (mm)	1.0x1.0x1.2				0.49x0.49x2.99	0.65x0.65x3.0
	n	16				16	16
**Basel** (n=203)	*Type*	NA	/	NA	NA	3D-FLAIR	3D-EPI T2*
	Resolution (mm)		1.0x1.0x1.0			1.0x1.0x1.0	0.67x0.67x0.67
	n		203			203	203
**Brussels** (n=25)	*Type*	NA	/	/	NA	3D-FLAIR	3D-EPI T2*
	Resolution (mm)		1.0x1.0x1.0	1.3x0.5x0.5		1.0x1.0x1.0	0.67x0.67x0.67
	n		25	25		25	25
**EPAD** (n=84)	*Type*	/	NA	NA	NA	3D-FLAIR	SWI
	Resolution (mm)	1.06x1.06x1.2				1.06x1.06x1.2	0.57x0.57x2.0
	n	84				84	84
**Graz** (n=34)	*Type*	/	NA	NA	NA	3D-FLAIR	3D-EPI T2*
	Resolution (mm)	1.0x1.0x1.0				1.0x1.0x1.0	0.65x0.65x0.65
	n	34				34	34
**Hannover** (n=34)	*Type*	/	/	/	NA	3D-FLAIR	SWI
	Resolution (mm)	0.9x1.0x1.0	1.0x1.0x1.0	1.2x1.0x1.0		0.9x0.5x0.5	0.5x0.5x3.0
	n	17	17	17		34	34
**Lausanne** (n=96)	*Type*	NA	/	/	NA	3D-FLAIR	SWI
	Resolution (mm)		1.2x1.0x1.0	1.2x1.0x1.0		1.2x0.5x0.5	0.69x0.69x1.4
	n		96	62		96	96
**London** (n=20)	*Type*	/	NA	NA	/	Axial PD-T2 TSE	SWI
	Resolution (mm)	1.0x1.0x1.0			0.5x0.5x2.0	1.0x1.0x3.0	1.0x1.0x1.0
	n	20			20	20	20
**Milan #1** (n=71)	*Type*	/	NA	/	NA	3D-FLAIR	SWI
	Resolution (mm)	1.0x1.0x1.0		0.89x0.89x1.0		0.89x0.89x1.0	0.60x0.60x1.0
	n	71		45		71	71
**Milan #2** (n=11)	*Type*	/	NA	NA	NA	3D-FLAIR	SWI
	Resolution (mm)	0.8x0.5x0.5				1.0x0.94x0.94	0.55x0.54x0.54
	n	11				11	11
**Naples #1** (n=113)	*Type*	/	NA	NA	NA	3D-FLAIR	3D SPGR
	Resolution (mm)	1.0x1.0x1.0				1.0x1.0x1.0	0.5x0.5x1.0
	n	113				113	113
**Naples #2** (n=17)	*Type*	/	NA	NA	NA	3D-FLAIR	3D SPGR
	Resolution	0.8x0.8x0.8				1.0x1.0x1.0	0.5x0.5x1.0

© 2023 Cagol A et al. *JAMA Neurology.*

	(mm)						
	n	17				17	17
**Oslo** (n=89)	*Type*	/	NA	NA	NA	3D-FLAIR	SWI
	Resolution (mm)	0.5x0.5x0.8				0.94x0.94x1.2	0.47x0.47x2.0
	n	89				89	89
**Oxford** (n=23)	*Type*	/	NA	/	NA	3D-FLAIR	SWI
	Resolution (mm)	1.0x1.0x1.0		1.3x1.3x1.3		1.0x1.0x1.0	1.3x1.3x1.3
	n	23		23		23	23
**Siena** (n=84)	*Type*	/	NA	/	NA	3D-FLAIR	SWI
	Resolution (mm)	1.0x1.0x1.0		1.0x1.0x3.0		0.98x0.98x1.0	0.3x0.3x1.0
	n	84		78		84	84
**Verona** (n=48)	*Type*	/	NA	/	NA	3D-FLAIR	3D-EPI T2*
	Resolution (mm)	1.0x1.0x1.0		1.0x1.0x1.0		1.0x1.0x1.0	0.55x0.55x0.55
	n	48		48		48	48

Abbreviations: 3D-T1, three dimensional T1-weighted; MP2RAGE, magnetization-prepared 2 rapid acquisition gradient echo; DIR, double inversion recovery; PSIR, phase-sensitive inversion recovery; FLAIR, fluid-attenuated inversion recovery; GRE, gradient echo; NA, not available; SWI, susceptibility-weighted imaging; EPI, echo-planar imaging.

© 2023 Cagol A et al. *JAMA Neurology.*

eTable 2. Comparison of the clinical, demographics, and MRI characteristics of the cohort with <2 years of disease duration and the entire cohort

	**Early CIS/MS cohort** (n=206)	**Entire CIS/MS cohort** (n=599)	**Comparison** (p-value)
Female, No. (%)	127 (62)	386 (64)	.52[b]
Age, mean (SD), years	36.7 (11.5)	41.5 (12.3)	**<.001**[c]
Disease duration, median [IQR], years	0.5 [0.3-1.0]	5.2 [1.0-13.0]	**<.001**[d]
EDSS score, median [IQR]	2.0 [1.0-2.5]	2.0 [1.5-3.5]	**<.001**[d]
Disease phenotype, No. (%)<br>- CIS<br>- RRMS<br>- SPMS<br>- PPMS	 43 (21)<br>158 (77)<br>0 (0)<br>5 (2)	 49 (8)<br>462 (77)<br>62 (10)<br>26 (4)	 **<.001**[b]<br>0.90[b]<br>-<br>0.22[b]
Participants with presence of OCBs, No. (%)[a]	142 (86)	365 (87)	.74[b]
Centers, n	12	14	/
Participants excluded from CL assessment (poor MRI quality), No. (%)	2 (1)	2 (0)	.26[b]
T1-weighted sequence:<br>- 3D-T1, No. (%)<br>- MP2RAGE, No. (%)	 107 (52)<br>97 (48)	 239 (40)<br>358 (60)	**.002**[b]
Participants with ≥1 CL, No. (%)	101 (50)	352 (59)	**.015**[b]
CL count, median [IQR]	0 [0-3]	1 [0-4]	**.008**[d]
Participants with WMLs suitable for CVS assessment, No. (%)	198 (96)	583 (97)	.38[b]
Susceptibility-based sequence:<br>- SWI, No. (%)<br>- T2*, No. (%)	 109 (55)<br>89 (45)	 318 (55)<br>265 (45)	.90[b]
Number of WMLs suitable for CVS assessment per participant, median [IQR]	8 [4-18]	11 [5-21]	**<.001**[d]
Percentage of CVS-positive lesions, median [IQR]	66.7 [50.0-84.1]	62.5 [44.2-80.0]	0.14[d]

Abbreviations: SD, standard deviation; IQR, interquartile range; CIS, clinically isolated syndrome; RRMS, relapsing-remitting multiple sclerosis; SPMS, secondary progressive multiple sclerosis; PPMS, primary progressive multiple sclerosis; OCBs, oligoclonal bands; MPRAGE, magnetization-prepared rapid gradient-echo; MP2RAGE, magnetization-prepared 2 rapid gradient-echo; CL, cortical lesion; SWI, susceptibility-weighted imaging; WMLs, white matter lesions; CVS, central vein sign.

[a] data available for 165 participants in the Early MS/CIS cohort, and for 419 participants in the Entire MS/CIS cohort.

[b] Pearson's chi-squared test.

[c] Welch's t-test.

[d] Mann-Whitney U test.

© 2023 Cagol A et al. *JAMA Neurology.*

eFigure 1. Diagnostic performance of CLs, CVS, and their combination in the entire cohort and in the subgroup of patients with < 2 years of disease duration

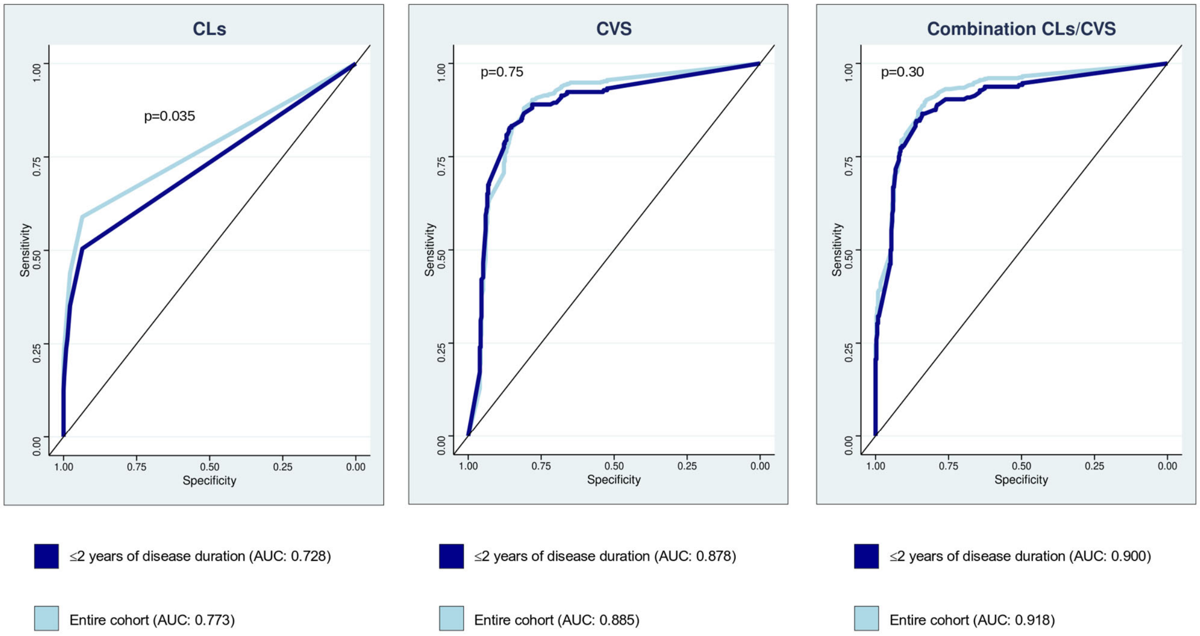

Entire cohort: n=934; including only MS/CIS patients with < 2 years of disease duration: n=657.
Abbreviations: CLs, cortical lesions; CVS, central vein sign; AUC, area under the curve.

© 2023 Cagol A et al. *JAMA Neurology.*

eFigure 2. Comparison of CVS diagnostic performance in the entire cohort vs in subjects with ≥3 lesions suitable for assessment

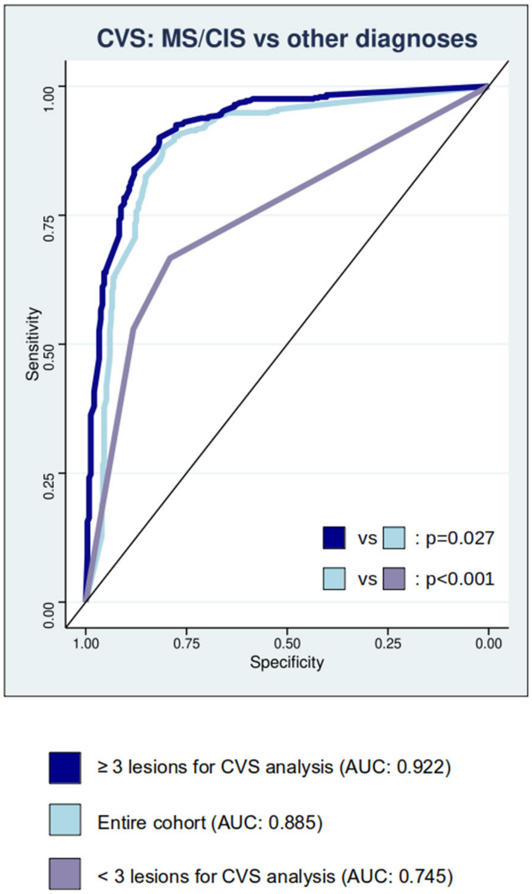

Entire cohort: n=934; subjects with ≥ 3 lesions suitable for CVS analysis: n=773; subjects with < 3 lesions suitable for CVS analysis: n=161.
Abbreviations: CVS, central vein sign; MS, multiple sclerosis; CIS, clinically isolated syndrome; AUC, area under the curve.

© 2023 Cagol A et al. *JAMA Neurology.*

eFigure 3. Comparison of CVS diagnostic performance: using a proportion-based threshold vs using the absolute number of CVS-positive lesions

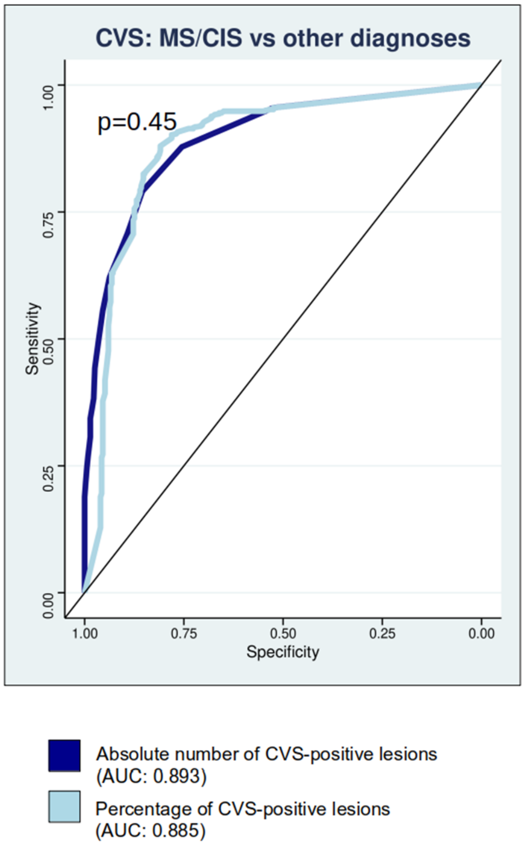

eTable 3: Sensitivity, specificity and accuracy for various absolute numbers of CVS-positive lesions

Threshold	Sensitivity	Specificity	Accuracy
1	95.5%	52.4%	79.3%
2	87.8%	75.5%	83.2%
3	79.1%	85.5%	81.5%
4	70.8%	89.2%	77.7%
5	62.1%	93.7%	74.0%
6	55.6%	95.4%	70.6%
7	49.2%	96.6%	67.0%
8	44.3%	97.4%	64.2%

© 2023 Cagol A et al. *JAMA Neurology.*

eTable 4. Prevalence of cortical lesion subtypes among different diagnosis groups

	IC	LC: GM>WM	LC: WM>GM	Curvilinear	Total
MS: No. (median; [IQR])	190 (0; [0-0])	594 (0; [0-1])	1442 (0; [0-2])	129 (0; [0-0])	2226 (1; [0-4])
CIS: No. (median; [IQR])	4 (0; [0-0])	42 (0; [0-2])	80 (0; [0-2])	3 (0; [0-0])	126 (0; [0-4])
AQP4-positive NMOSD: No. (median; [IQR])	0 (0; [0-0])	0 (0; [0-0])	1 (0; [0-0])	1 (0; [0-0])	1 (0; [0-0])
Seronegative NMOSD: No. (median; [IQR])	2 (0; [0-0])	4 (0; [0-0])	0 (0; [0-0])	3 (0; [0-0])	6 (0; [0-0])
MOGAD, No. (median; [IQR])	0 (0; [0-0])	3 (0; [0-0])	3 (0; [0-0])	2 (0; [0-0])	6 (0; [0-0])
Migraine, No. (median; [IQR])	1 (0; [0-0])	0 (0; [0-0])	0 (0; [0-0])	0 (0; [0-0])	1 (0; [0-0])
Inflammatory vasculopathies: No. (median; [IQR])	3 (0; [0-0])	6 (0; [0-0])	6 (0; [0-0])	0 (0; [0-0])	15 (0; [0-0])
Cerebrovascular disease: No. (median; [IQR])	3 (0; [0-0])	1 (0; [0-0])	13 (0; [0-0])	0 (0; [0-0])	17 (0; [0-0])
Fabry disease: No. (median; [IQR])	0 (0; [0-0])	0 (0; [0-0])	0 (0; [0-0])	0 (0; [0-0])	0 (0; [0-0])
Healthy controls: No. (median; [IQR])	0 (0; [0-0])	2 (0; [0-0])	4 (0; [0-0])	0 (0; [0-0])	6 (0; [0-0])

Abbreviations: IC, intracortical; LC: GM>WM, leukocortical with prevalent gray matter involvement; LC: WM>GM, leukocortical with prevalent white matter involvement; MS, multiple sclerosis; AQP-4, aquaporin-4 antibody; NMOSD, neuromyelitis optica spectrum disorder; MOGAD, myelin oligodendrocyte glycoprotein antibody-associated disease; IQR, interquartile range.

© 2023 Cagol A et al. *JAMA Neurology.*

eFigure 4. Examples of cortical lesions in patients with multiple sclerosis

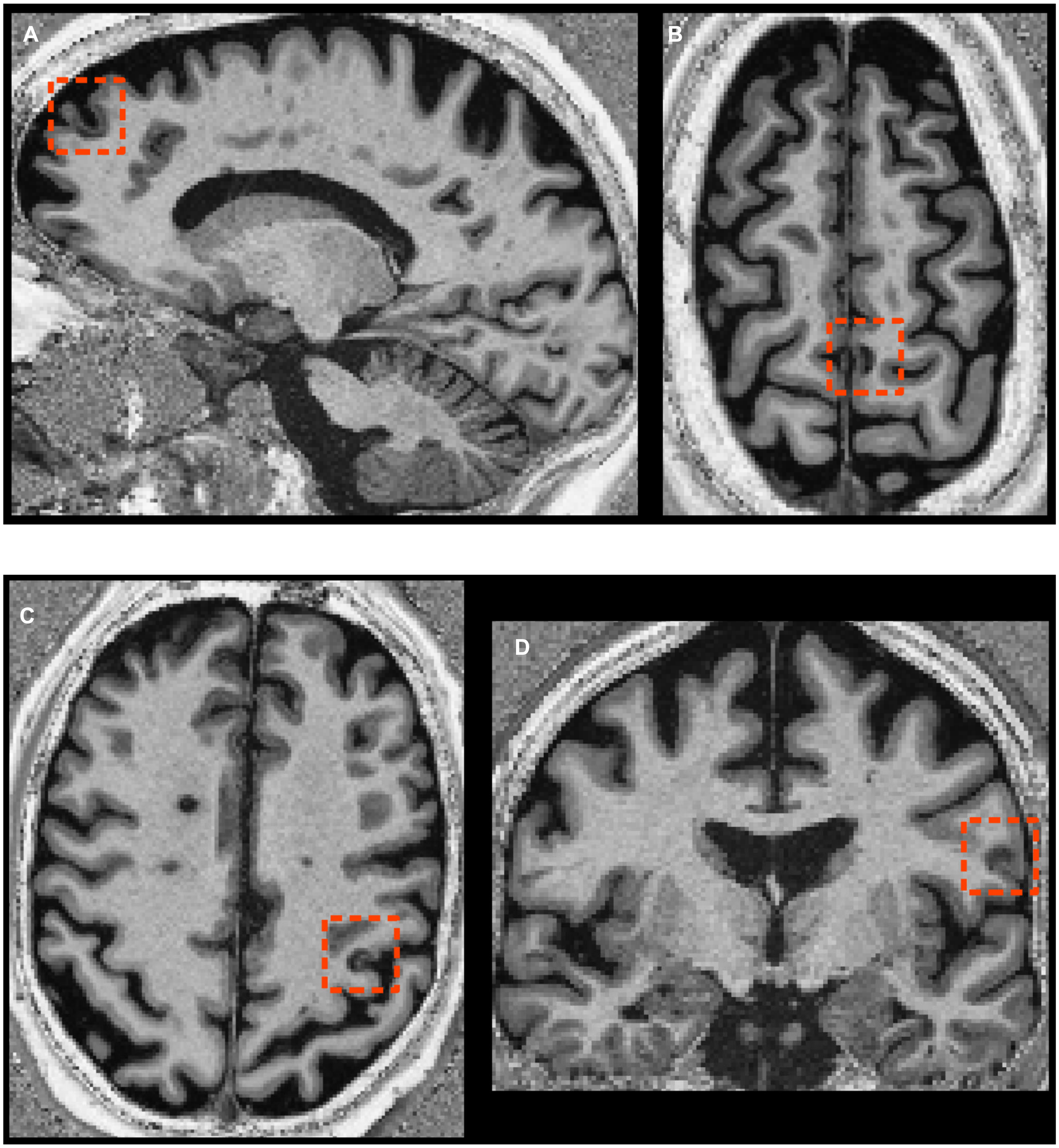

© 2023 Cagol A et al. *JAMA Neurology.*

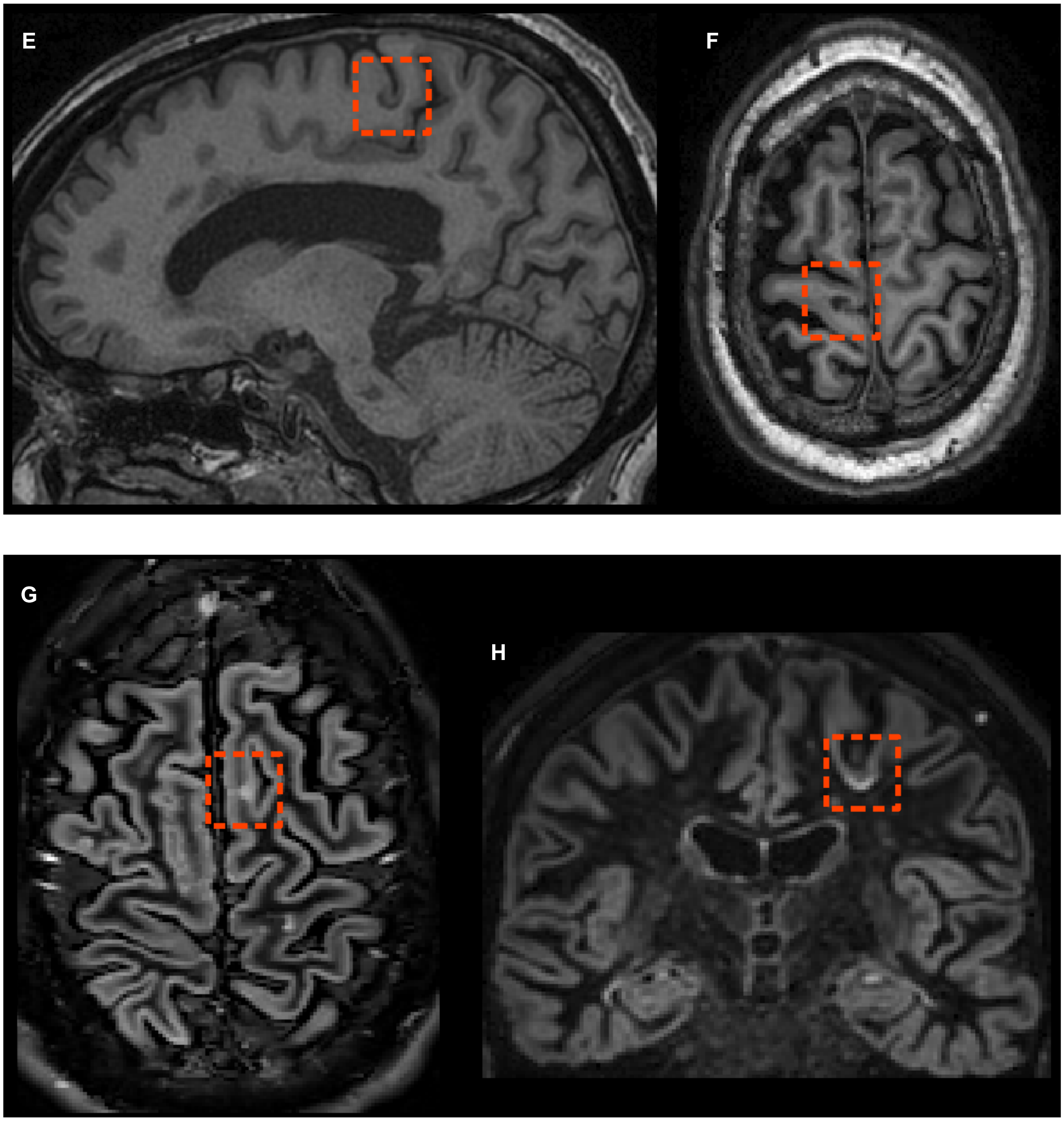

© 2023 Cagol A et al. *JAMA Neurology.*

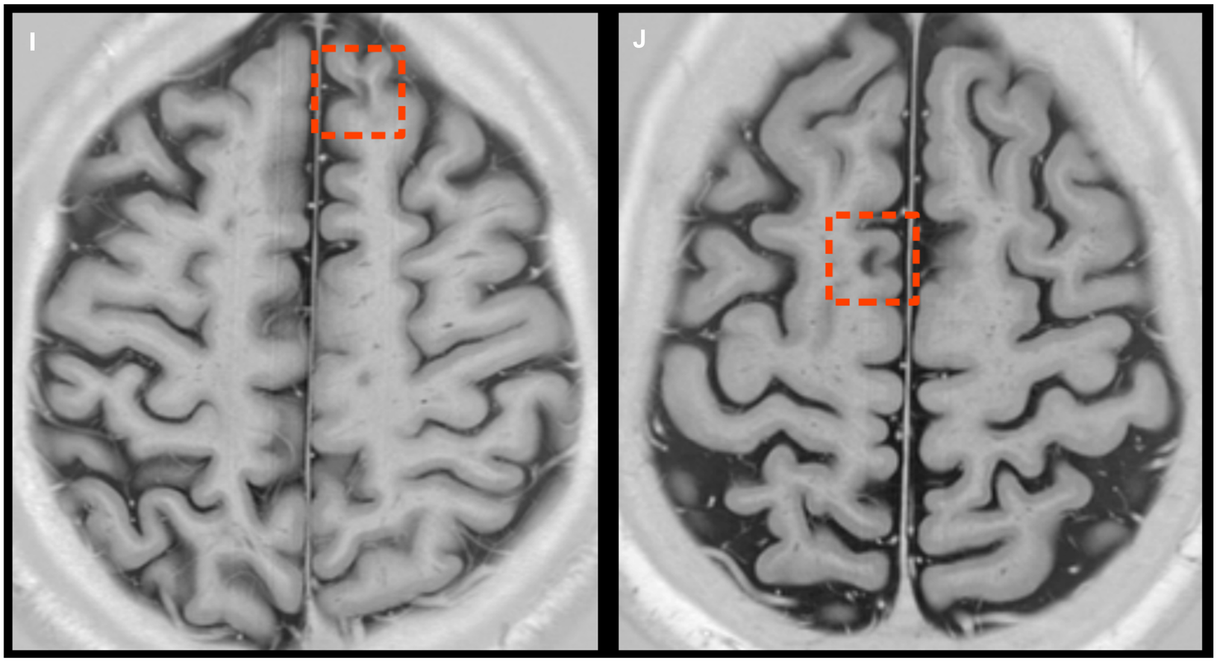

Panels A-D: MP2RAGE images.
Panels E-F: 3D-T1 images.
Panels G-H: DIR images.
Panels I-J: PSIR images.

© 2023 Cagol A et al. *JAMA Neurology.*

eFigure 5. Examples of cortical lesions in non-MS conditions

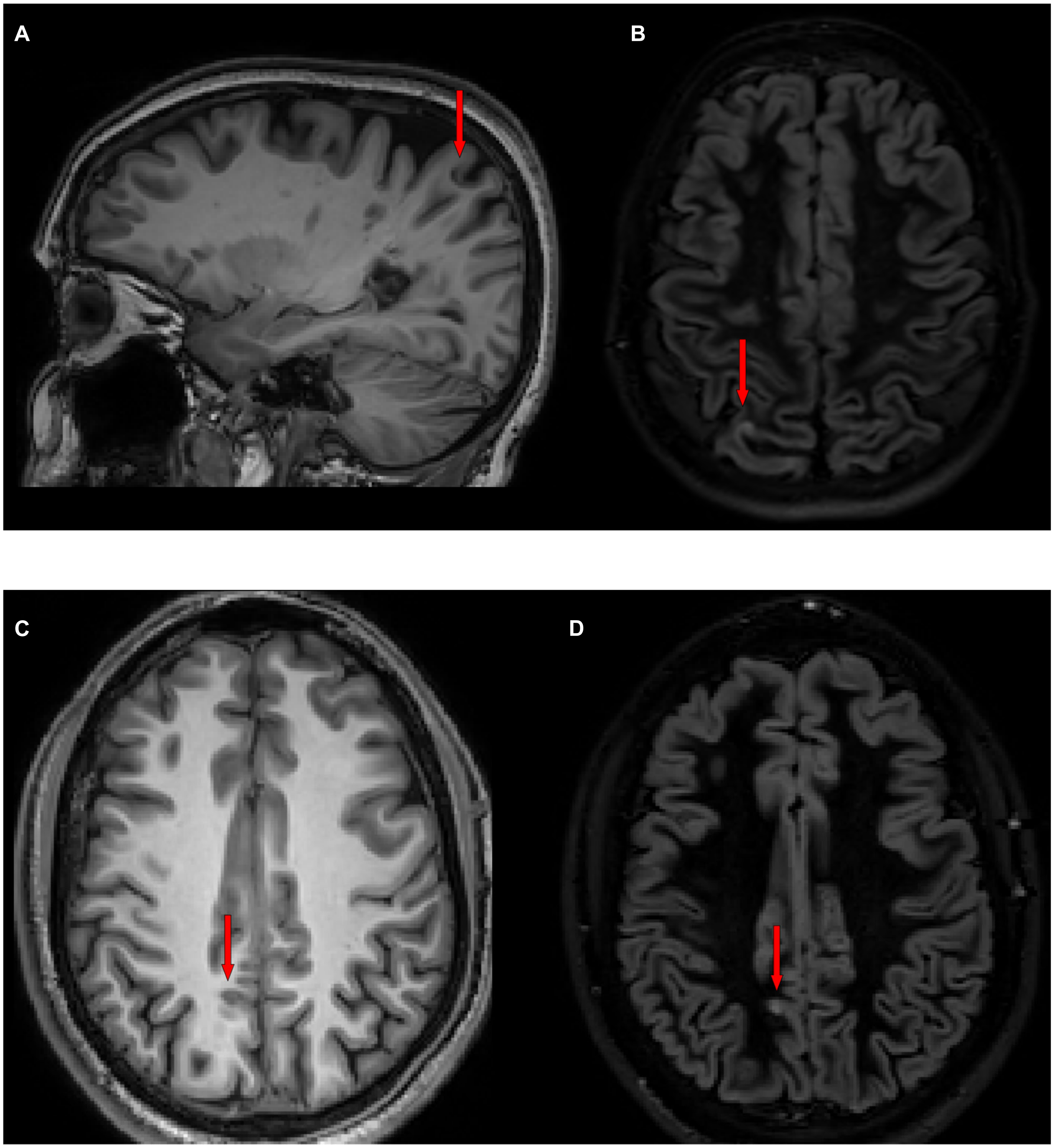

© 2023 Cagol A et al. *JAMA Neurology.*

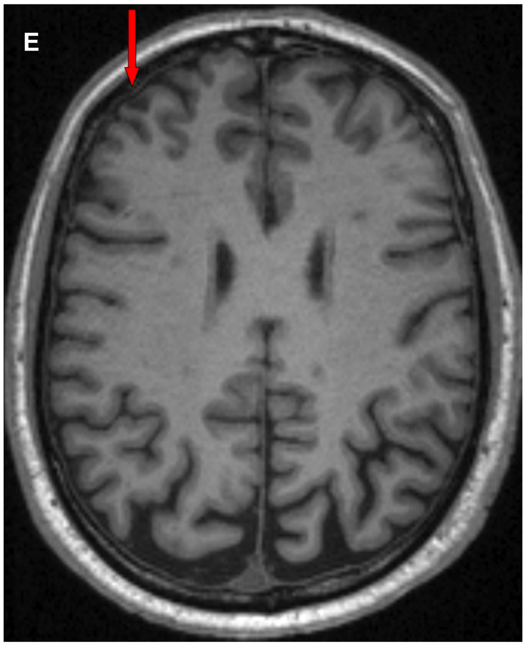

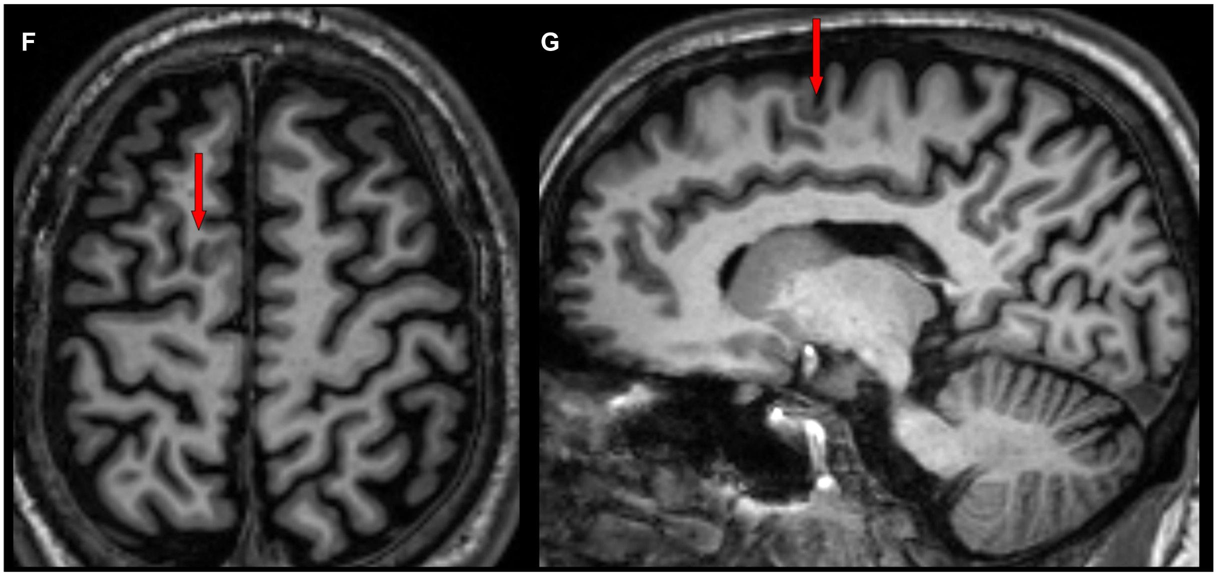

© 2023 Cagol A et al. *JAMA Neurology.*

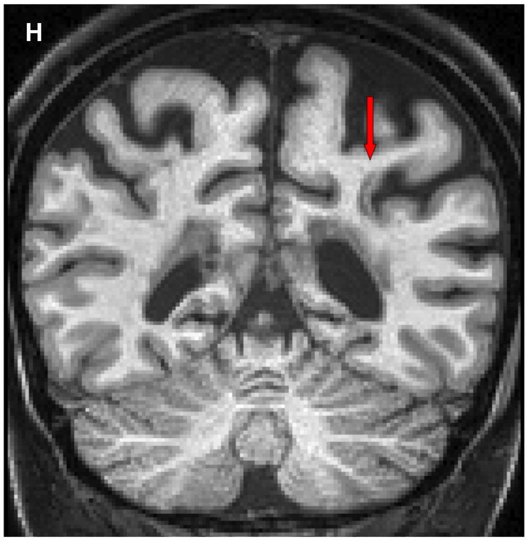

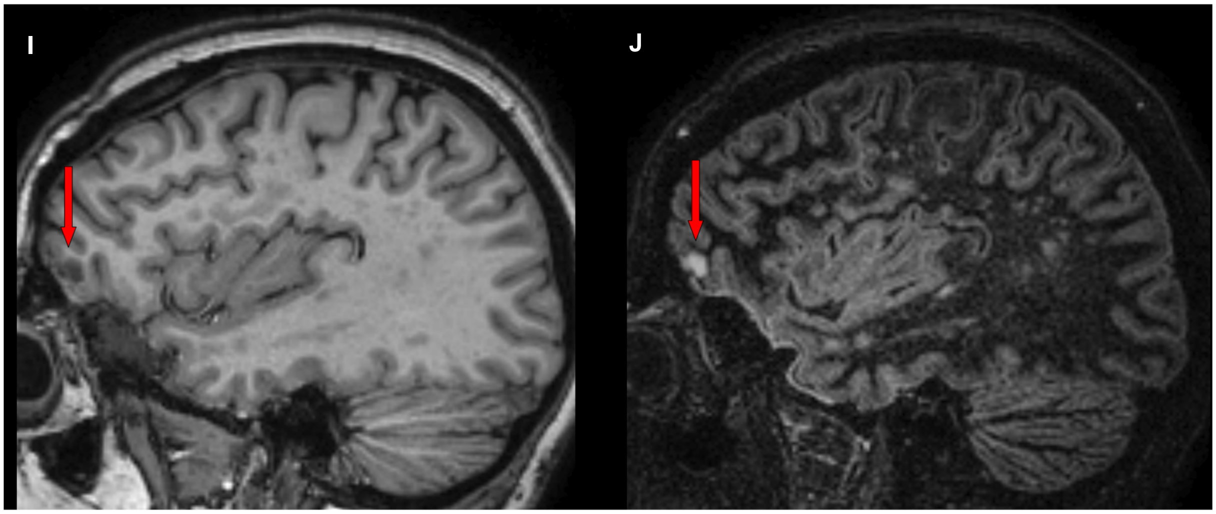

Examples of cortical lesions in: a 50 years old female with MOGAD (panels A and B); a 33 years old male with migraine (panels C and D); a 67 years old male with Sjögren syndrome (panel E); a 40 years old female with AQP4-positive NMOSD (panels F and G); a 56 years old male with seronegative NMOSD (panel H); a 50 years old female with cerebrovascular disease (panels I and J).
Panel A, C, E, F, G, H, and I: 3D-T1 images; panels B, D, and J: DIR images.

© 2023 Cagol A et al. *JAMA Neurology.*

eFigure 6. Examples of images used for CVS assessment

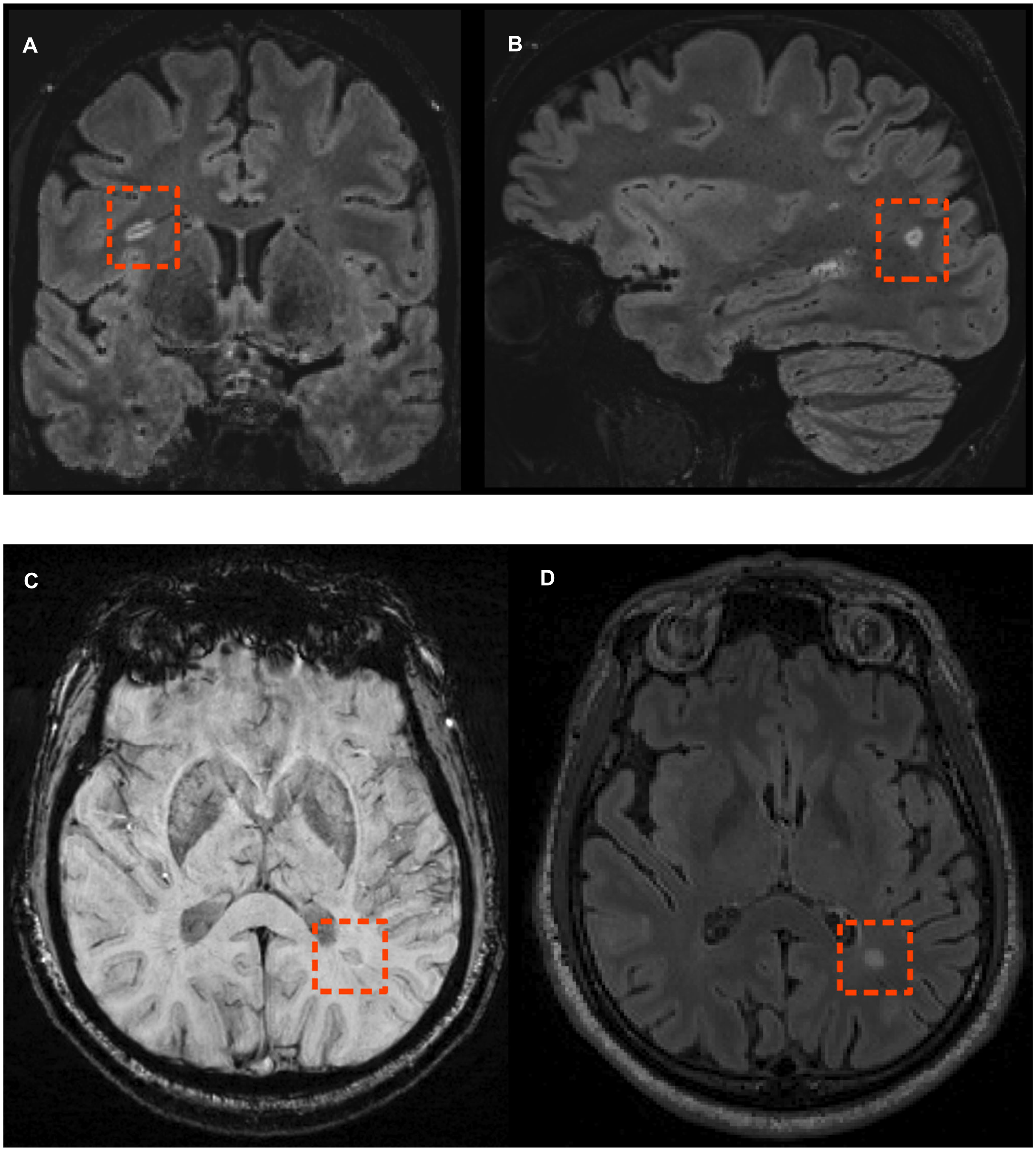

© 2023 Cagol A et al. *JAMA Neurology.*

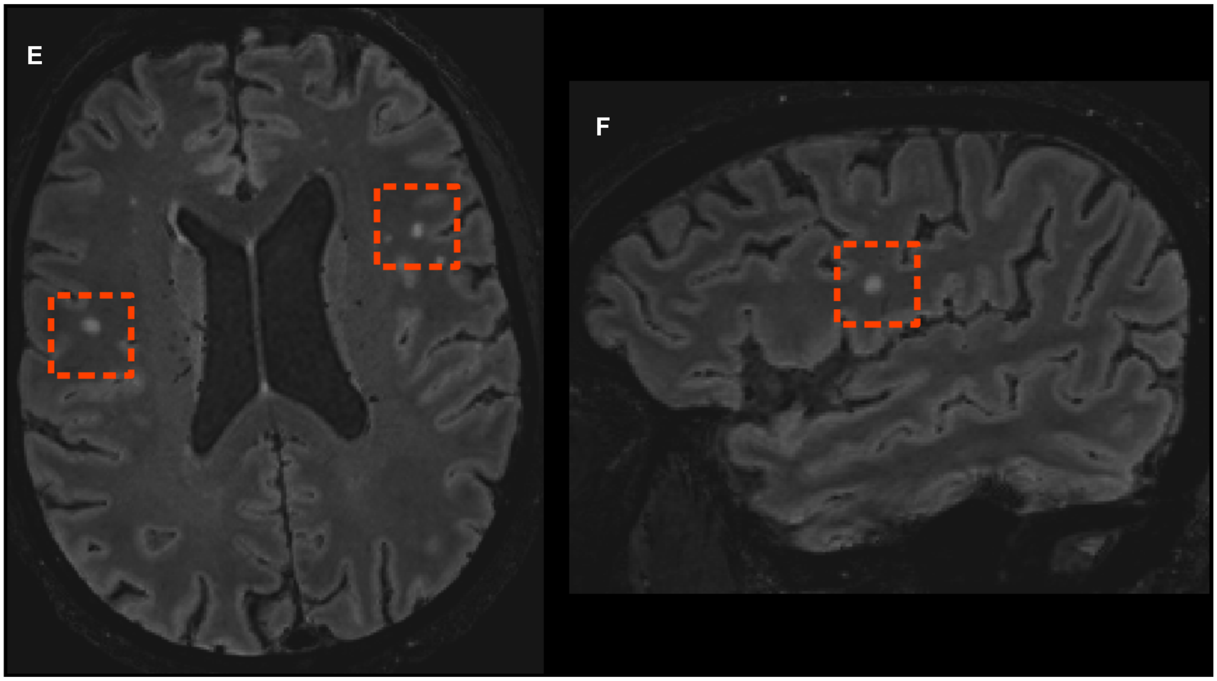

Panels A and B: examples of CVS-positive lesions on FLAIR* images in patients with multiple sclerosis.
Panel C: example of CVS-positive lesion on SWI in a patient with multiple sclerosis. The lesion is clearly visible on FLAIR (panel D).
Panels E and F: examples of CVS-negative lesions on FLAIR* images in healthy controls.

© 2023 Cagol A et al. *JAMA Neurology.*

eFigure 7. Proportion of CVS-positive lesions per location in MS/CIS and non-MS conditions

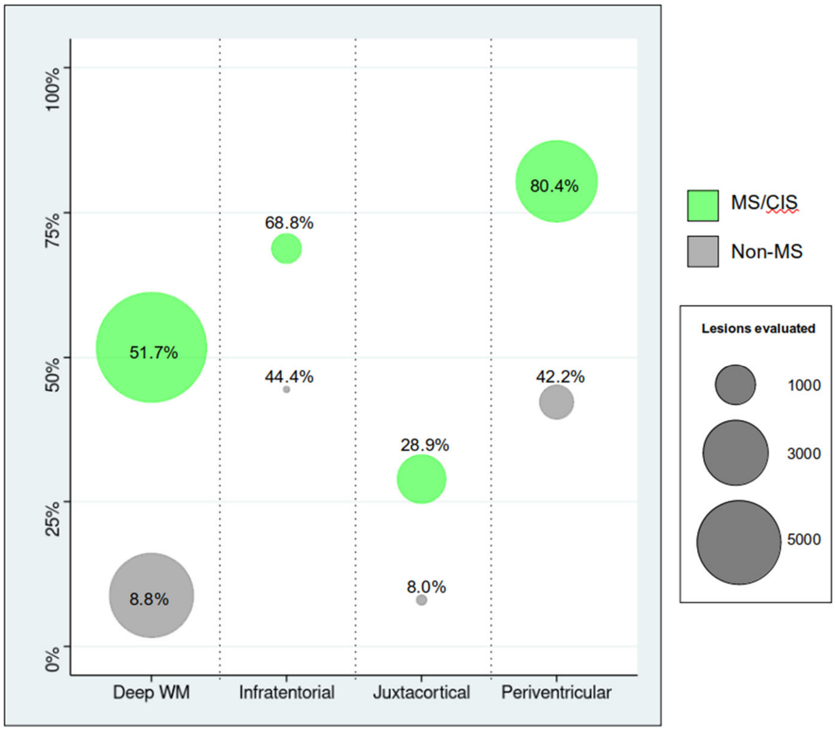

Abbreviations: WM, white matter; MS, multiple sclerosis; CIS, clinically isolated syndrome.

eFigure 8. Ratio of CVS-positive lesions between MS/CIS and non-MS conditions per location

Abbreviations: WM, white matter.

© 2023 Cagol A et al. *JAMA Neurology.*

eFigure 9. Examples of images used to blind raters to the general appearance of the scan

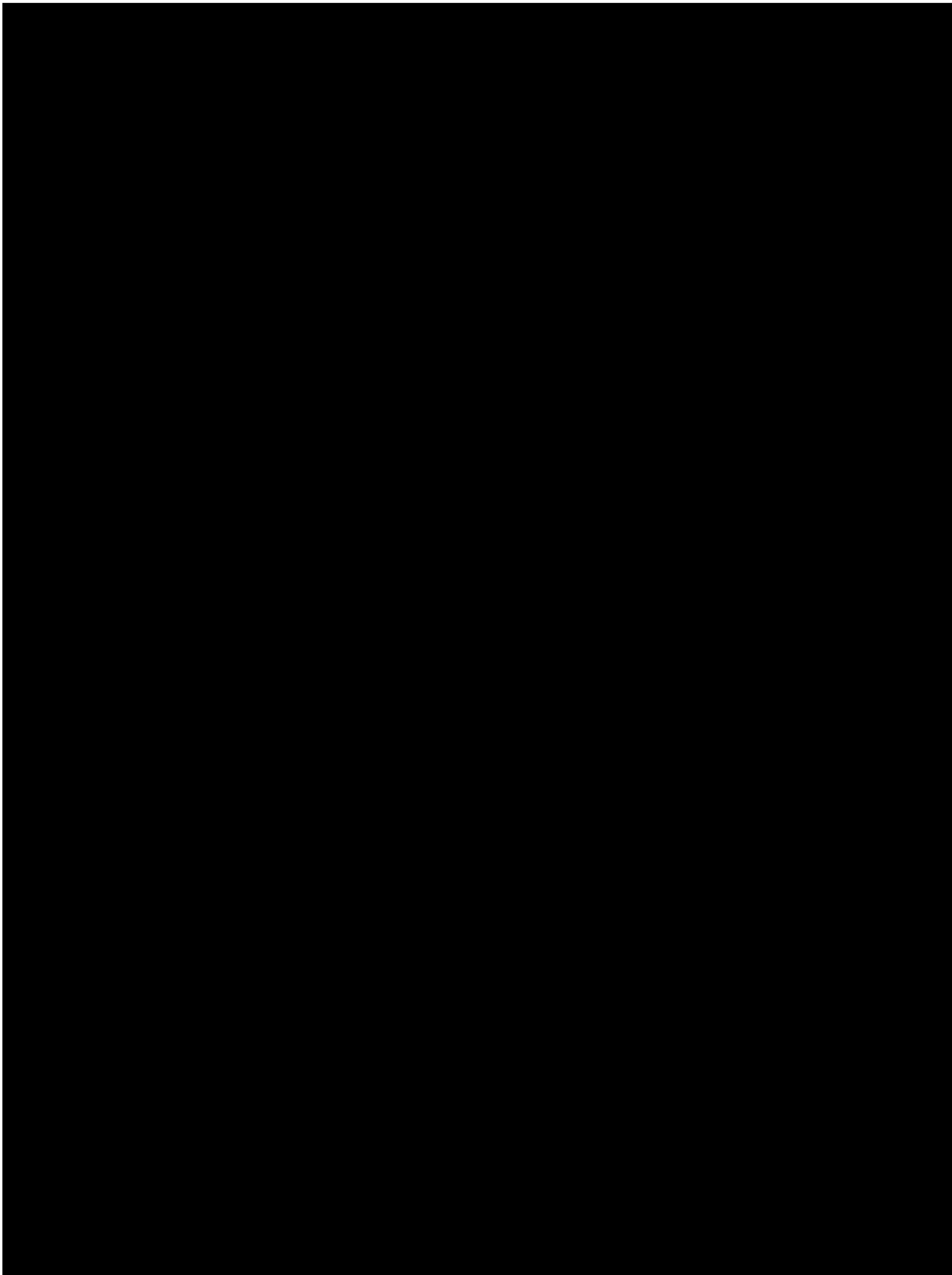

eTable 5. Diagnostic performance of the "Select-3" algorithm

Threshold	Sensitivity	Specificity	Accuracy
1	84.0	59.3	75.6
2	51.1	92.9	65.4
3	21.9	98.2	47.9
Participants: n=332; AUC: 0.794 (*95%-CI*: 0.748; 0.840)			

Abbreviations: AUC, area under the curve; CI, confidence interval.

eFigure 10. Comparison of the diagnostic performance of the "Select-3" algorithm and the proportion of CVS-positive lesions

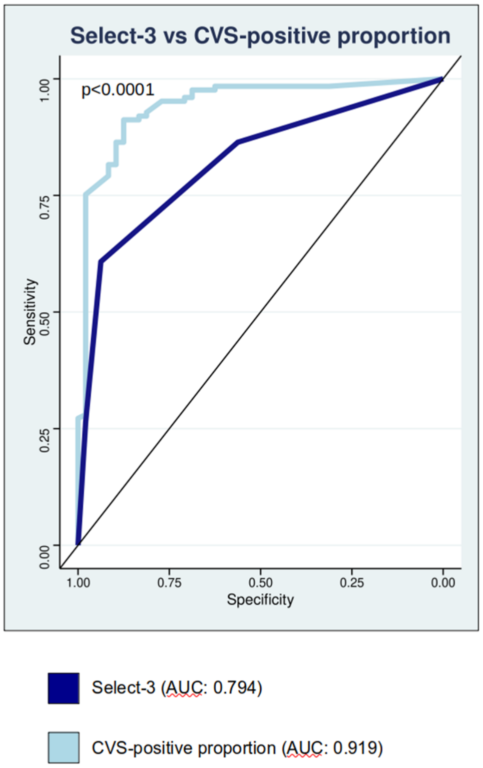

Abbreviations: CVS, central vein sign; AUC, area under the curve.

© 2023 Cagol A et al. *JAMA Neurology.*

eTable 6. Diagnostic performance of the “Pick-6” algorithm

Threshold	Sensitivity	Specificity	Accuracy
1	95.7	42.1	81.3
2	89.4	76.3	85.9
3	72.6	90.8	77.5
4	52.4	97.4	64.4
5	30.3	97.4	48.2
6	11.1	100	34.9
Participants: n=284; AUC: 0.892 (*95%-CI*: 0.851; 0.933)			

Abbreviations: AUC, area under the curve; CI, confidence interval.

eFigure 11. Comparison of the diagnostic performance of the “Pick-6” algorithm and the proportion of CVS-positive lesions

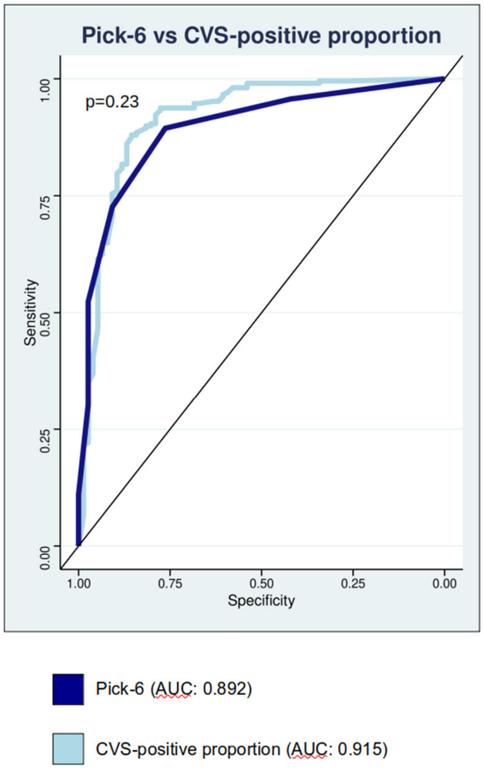

Abbreviations: CVS, central vein sign; AUC, area under the curve.

© 2023 Cagol A et al. *JAMA Neurology.*

eTable 7. Diagnostic performance of the "Select-n*" algorithm

Threshold	Sensitivity	Specificity	Accuracy
Select-1*	96.4	30.4	84.2
Select-2*	90.7	57.1	84.5
Select-3*	83.1	64.3	79.6
Select-4*	76.2	71.4	75.3
Select-5*	69.4	85.7	72.4
Select-6*	62.5	92.9	68.1
Participants: n=310; AUC: 0.855 (*95%-CI*: 0.808; 0.902)			

Threshold	Sensitivity	Specificity	Accuracy
Select-6*[2]	82.7	87.5	83.6

Abbreviations: AUC, area under the curve; CI, confidence interval.

eFigure 12. Comparison of the diagnostic performance of the "Select-n*" algorithm and the proportion of CVS-positive lesions

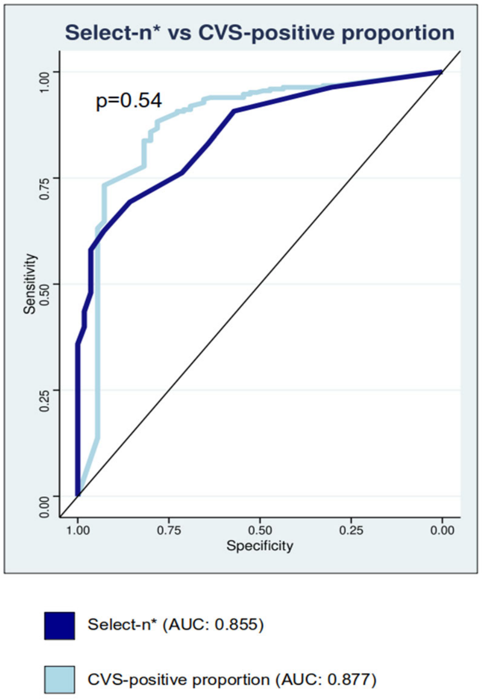

Abbreviations: CVS, central vein sign; AUC, area under the curve.

© 2023 Cagol A et al. *JAMA Neurology.*

eReferences

1. La Rosa F, Abdulkadir A, Fartaria MJ, Rahmanzadeh R, Lu PJ, Galbusera R, et al. Multiple sclerosis cortical and WM lesion segmentation at 3T MRI: a deep learning method based on FLAIR and MP2RAGE. NeuroImage Clin [Internet]. 2020 Jan 1 [cited 2021 Nov 24];27. Available from: https://pubmed.ncbi.nlm.nih.gov/32663798/

2. Filippi M, Preziosa P, Banwell BL, Barkhof F, Ciccarelli O, De Stefano N, et al. Assessment of lesions on magnetic resonance imaging in multiple sclerosis: practical guidelines. Vol. 142, Brain. 2019. p. 1858–75.

3. Geurts JJG, Roosendaal SD, Calabrese M, Ciccarelli O, Agosta F, Chard DT, et al. Consensus recommendations for MS cortical lesion scoring using double inversion recovery MRI. Neurology [Internet]. 2011 Feb 1 [cited 2023 Mar 1];76(5):418–24. Available from: https://n.neurology.org/content/76/5/418

4. Sethi V, Yousry TA, Muhlert N, Ron M, Golay X, Wheeler-Kingshott C, et al. Improved detection of cortical MS lesions with phase-sensitive inversion recovery MRI. J Neurol Neurosurg Psychiatry [Internet]. 2012 [cited 2023 Mar 1];83(9):877–82. Available from: https://pubmed.ncbi.nlm.nih.gov/22807559/

5. Calabrese M, Battaglini M, Giorgio A, Atzori M, Bernardi V, Mattisi I, et al. Imaging distribution and frequency of cortical lesions in patients with multiple sclerosis. Neurology [Internet]. 2010 [cited 2022 Nov 17];75(14):1234–40. Available from: www.fmrib.ox.ac.uk/fsl/

6. Isensee F, Schell M, Pflueger I, Brugnara G, Bonekamp D, Neuberger U, et al. Automated brain extraction of multisequence MRI using artificial neural networks. Hum Brain Mapp [Internet]. 2019 Dec 1 [cited 2022 Nov 17];40(17):4952–64. Available from: https://onlinelibrary.wiley.com/doi/full/10.1002/hbm.24750

7. Avants BB, Tustison NJ, Song G, Cook PA, Klein A, Gee JC. A reproducible evaluation of ANTs similarity metric performance in brain image registration. Neuroimage [Internet]. 2011 Feb 1 [cited 2022 Nov 17];54(3):2033–44. Available from: https://pubmed.ncbi.nlm.nih.gov/20851191/

8. Sati P, George IC, Shea CD, Gaitán MI, Reich DS. FLAIR*: A combined MR contrast technique for visualizing white matter lesions and parenchymal veins. Radiology [Internet]. 2012 [cited 2022 Nov 17];265(3):926–32. Available from: https://pubmed.ncbi.nlm.nih.gov/23074257/

9. Puonti O, Iglesias JE, Van Leemput K. Fast and sequence-adaptive whole-brain segmentation using parametric Bayesian modeling. Neuroimage. 2016 Dec 1;143:235–49.

10. Koo TK, Li MY. A Guideline of Selecting and Reporting Intraclass Correlation Coefficients for Reliability Research. J Chiropr Med [Internet]. 2016 Jun 1 [cited 2022 Dec 6];15(2):155–63. Available from: /pmc/articles/PMC4913118/

11. Sun X, Xu W. Fast implementation of DeLong's algorithm for comparing the areas under correlated receiver operating characteristic curves. IEEE Signal Process Lett. 2014;21(11):1389–93.

12. Solomon AJ, Watts R, Ontaneda D, Absinta M, Sati P, Reich DS. Diagnostic performance of central vein sign for multiple sclerosis with a simplified three-lesion algorithm. Mult Scler J [Internet]. 2018 May 1 [cited 2023 Sep 24];24(6):750–7. Available from: https://pubmed.ncbi.nlm.nih.gov/28820013/

13. Cortese R, Magnollay L, Tur C, Abdel-Aziz K, Jacob A, De Angelis F, et al. Value of the central vein sign at 3T to differentiate MS from seropositive NMOSD. Neurology [Internet]. 2018 [cited 2023 Sep 24];90(14):e1183–90. Available from: https://pubmed.ncbi.nlm.nih.gov/29514948/

14. Chaaban L, Safwan N, Moussa H, El-Sammak S, Khoury SJ, Hannoun S. Central vein sign: A putative diagnostic marker for multiple sclerosis. Vol. 145, Acta Neurologica Scandinavica. John Wiley and Sons Inc; 2022. p. 279–87.

15. Ontaneda D, Sati P, Raza P, Kilbane M, Gombos E, Alvarez E, et al. Central vein sign: A diagnostic biomarker in multiple sclerosis (CAVS-MS) study protocol for a prospective multicenter trial. NeuroImage Clin [Internet]. 2021 Jan 1 [cited 2023 Sep 24];32. Available from: /pmc/articles/PMC8482479/

16. Daboul L., O'Donnell C., Moreno-Dominguez D., Rodrigues P., Derbyshire J., Azevedo C., Bar-Or A., Caverzasi E., Calabresi P., Cree B., Freeman L., Henry R., Longbrake E., Oh J., Papinutto N., Pelletier D., Samudralwar R., Schindler M., Sotirchos E., Sicotte N., Ontaneda D. Gadolinium improves detection of central vein lesions in MS using 3t flair*. MSVirtual 2020 – Poster Abstracts. Mult. Scler. J. 2020;2020(26):118–659.

17. Mistry N, Abdel-Fahim R, Samaraweera A, Mougin O, Tallantyre E, Tench C, et al. Imaging central veins in brain lesions with 3-T T2∗-weighted magnetic resonance imaging differentiates multiple sclerosis from microangiopathic brain lesions. Mult Scler [Internet]. 2016 Sep 1 [cited 2023 Mar 30];22(10):1289–96. Available from: https://pubmed.ncbi.nlm.nih.gov/26658816/

© 2023 Cagol A et al. *JAMA Neurology.*
