# Supplementary material for: Diagnostic Performance of Cortical Lesions and the Central Vein Sign in Multiple Sclerosis
Source: JAMA Neurol. 2023 Dec 11;81(2):143–53. doi: 10.1001/jamaneurol.2023.4737 (PMC10714285; doi:10.1001/jamaneurol.2023.4737)
Supplement: Supplement 2. — Members of the Magnetic Resonance Imaging in MS (MAGNIMS) Study Group [file jamaneurol-e234737-s002.pdf]

Supplemental Online Content: Nonauthor Collaborators
*First name, last name, and suffix (if applicable) are required and will appear in PubMed.

*Group Name(s): Magnetic Resonance Imaging in MS (MAGNIMS) Study Group							
***First Name and Middle Initial(s)**	***Last Name**	***Suffix (eg, Jr, III)**	Academic Degrees	Institution	Location (city, state/province, country)	Role or Contribution, eg, chair, principal investigator	Group (if more than 1 Group listed in the byline) and/or Subgroup (eg, Steering Committee)
Jaume	Sastre-Garriga		MD, PhD	Multiple Sclerosis Centre of Catalonia, Department of Neurology/Neuroimmunology, Hospital Universitari Vall d'Hebron, Universitat Autònoma de Barcelona, Barcelona, Spain	Barcelona, Spain	Co-chair of the MAGNIMS Steering Committee	MAGNIMS Steering Committee
Claudio	Gasperini		MD	Department of Neurology, San Camillo-Forlanini Hospital, Rome, Italy	Rome, Italy	Member of the MAGNIMS Steering Committee	MAGNIMS Steering Committee
Hugo	Vrenken		PhD	MS Center Amsterdam, Radiology and Nuclear Medicine, Vrije Universiteit Amsterdam, Amsterdam Neuroscience, Amsterdam UMC Location VUmc, Amsterdam, The Netherlands	Amsterdam, The Netherlands	Member of the MAGNIMS Steering Committee	MAGNIMS Steering Committee
Tarek	Yousry		MD	Lysholm Department of Neuroradiology, UCLH National Hospital for Neurology and Neurosurgery, Neuroradiological Academic Unit, UCL Institute of Neurology, London, UK	London, UK	Member of the MAGNIMS Steering Committee	MAGNIMS Steering Committee
