# Supplementary material for: Diagnostic Performance of Cortical Lesions and the Central Vein Sign in Multiple Sclerosis
Source: JAMA Neurol. 2023 Dec 11;81(2):143–53. doi: 10.1001/jamaneurol.2023.4737 (PMC10714285; doi:10.1001/jamaneurol.2023.4737)
Supplement: Supplement 3. — Data sharing statement [file jamaneurol-e234737-s003.pdf]

Data Sharing Statement

Data
Data available: No

Additional Information
Explanation for why data not available: The data that support the findings of this study are controlled by the respective centers, and are not publicly available. Request to access the raw data should be forwarded to data controllers via the corresponding author. Written requests for access to the derived data will be considered by the corresponding author and a decision made about the appropriateness of the use of the data.
